# Cell‐Surface LAMP1 is a Senescence Marker in Aging and Idiopathic Pulmonary Fibrosis

**DOI:** 10.1111/acel.70141

**Published:** 2025-06-22

**Authors:** Gabriel Meca‐Laguna, Michael Qiu, Yafei Hou, Anna Barkovskaya, Apoorva Shankar, Bhavna Dixit, Michael J. Rae, Amutha Boominathan, Amit Sharma

**Affiliations:** ^1^ Lifespan Research Institute Mountain View California USA; ^2^ Department of Biochemistry and Molecular Biology University of Miami Miller School of Medicine Miami Florida USA

**Keywords:** ADC, aging, biomarker, LAMP1, senescence, surface

## Abstract

The accumulation of senescent cells (SEN) with aging produces a chronic inflammatory state that accelerates age‐related diseases. Eliminating SEN has been shown to delay, prevent, and in some cases reverse aging in animal disease models and extend lifespan. There is thus an unmet clinical need to identify and target SEN while sparing healthy cells. Here, we show that Lysosomal‐Associated Membrane Protein 1 (LAMP1) is a membrane‐specific biomarker of cellular senescence. We have validated selective LAMP1 upregulation in SEN in human and mouse cells. Lamp1^+^ cells express high levels of the prototypical senescence markers p16, p21, Glb1, and have low Lmnb1 expression as compared to Lamp1^−^ cells. The percentage of Lamp1^+^ cells is increased with age and in mice with fibrotic lungs due to bleomycin (BLM) instillation. The RNA‐Sequencing analysis of the Lamp1‐enriched populations in sham and BLM mice lung tissue revealed enrichment of several senescence‐related genes in both groups when compared to the SenMayo gene set derived from transcriptomic profiling of senescence markers in Mayo Clinic research datasets. Finally, we use a dual antibody‐drug conjugate (ADC) strategy to eliminate SEN in cell culture assay.

## Graphical abstract

We have validated the selective upregulation of LAMP1 in SEN in culture, and the percentage of Lamp1^+^ cells increases in various tissues with age, as well as in fibrotic lungs following BLM instillation. The RNA‐Sequencing analysis of the Lamp1‐enriched populations confirmed the enrichment of senescence‐related genes.

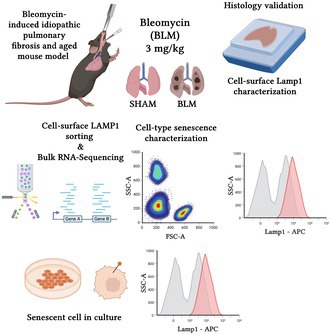

## 1 Introduction

Throughout life, cells are continually exposed to a myriad of different stressors, including oncogenic stress, telomeric shortening, wounds, infections, irradiation, and certain drugs. If the cellular insult constitutes irreparable damage, programmed cell death pathways provoke apoptosis in the affected cells. However, most such challenges can be resolved, in which case damaged cells arrest their cycle to restore homeostatic conditions eventually. Cellular senescence, initially described in 1961 by Leonard Hayflick (Hayflick and Moorhead 1961), is an irreversible cell cycle arrest following cellular insult (Munoz‐Espin and Serrano 2014). Phenotypically, senescent cells (SEN) secrete a cocktail of pro‐inflammatory and other molecules known as the senescence‐associated secretory phenotype (SASP), which contributes to the chronic, sterile, low‐grade inflammation of aging (inflammaging), accelerating morbidity onset (Munoz‐Espin and Serrano 2014; Xu et al. 2018). Accordingly, transplantation of SEN induces physical dysfunction in mice (Xu et al. 2018). Conversely, eliminating SEN has been shown to increase health and lifespan (Kirkland and Tchkonia 2017). Thus, SEN contribute to age‐related decline. For this reason, there is a growing interest in developing interventions that eliminate SEN or modulate their inflammatory secretory phenotype (Kirkland and Tchkonia 2017).

So far, the combination of dasatinib and quercetin (D+Q), navitoclax, and fisetin are the most widely tested senolytics, which target apoptotic vulnerabilities in SEN (Chang et al. 2016; Yousefzadeh et al. 2018; Zhu et al. 2015). Additionally, the immune system executes surveillance on SEN (Kale et al. 2020). Investigators have exploited chimeric antigen receptor (CAR) biotechnology to target SEN with engineered lymphocytes, leveraging surface proteins like uPAR or stress‐associated NKG2D ligands (Amor et al. 2020; Deng et al. 2024; Yang et al. 2023). Approaches with small molecules have shown promising results in vitro and in vivo in various models. However, limited efficacy has been observed in human clinical trials (Nambiar et al. 2023) or in genetically heterogeneous mice in the Interventions Testing Program (ITP) (Harrison et al. 2024). Other strategies that have been used to target SEN in vitro and in vivo based on their surface proteome (surfaceome) include antibody‐drug conjugates (ADCs) against B2M and ApoD (Poblocka et al. 2021; Takaya et al. 2023) and antibody‐dependent cell‐mediated cytotoxicity (ADCC) against DPP4 (Kim et al. 2017).

Despite the well‐documented role of SEN in aging and age‐related diseases, the lack of a robust biomarker for characterizing senescence in tissue samples is a significant impediment in the field. Such a biomarker should be agnostic to the tissue and cell type of origin, as well as the senescence‐causing insult. The lack of such a biomarker limits researchers' ability to evaluate SEN elimination (senolysis) after candidate senolytic treatments.

One of the most well‐documented hallmarks of SEN is their increased lysosomal content and activity (Dimri et al. 1995; Hernandez‐Segura et al. 2017; Rovira et al. 2022). This hallmark feeds into multiple other features of senescence, such as changes in morphology, the SASP phenotype, and metabolic alterations. Indeed, early efforts quantifying lysosomal activity resulted in the discovery of senescence‐associated β‐galactosidase (SA‐β‐Gal), one of the most widely used biomarkers of senescence (Dimri et al. 1995).

Lysosomal‐Associated Membrane Protein 1 (LAMP1, also known as CD107a) is a master orchestrator of the structural integrity of lysosomes (Eskelinen 2006). It is estimated that LAMP1 (and LAMP2) constitute half of all proteins of the lysosomal membrane (Eskelinen 2006; Hunziker and Geuze 1996). LAMP1, as a type I transmembrane glycoprotein, is mostly localized in late endosomes and lysosomes (Hunziker and Geuze 1996) and is a heavily N‐glycosylated protein (Alessandrini et al. 2017). In immune cells, CD107a is also a cell‐surface marker of immune activation and cytotoxic degranulation (Alessandrini et al. 2017; Krzewski et al. 2013); however, it can only be detected using inhibitors of intracellular protein transport (such as GolgiStop^TM^, monesin‐containing, or brefeldin A‐containing reagents) since its expression on the membrane is transient and the protein is internalized rapidly. Therefore, LAMP1 is only briefly found at the cell surface of healthy cells due to the fusion of lysosomes with the plasma membrane, and thus, is mostly undetectable. In the context of disease, it has been reported that melanoma, carcinoma, and fibrosarcoma cells exhibit abnormal localization of LAMP1 on their plasma membranes, possibly as part of the repair of the damaged cell surface (Corrotte et al. 2015; Reddy et al. 2001; Sarafian et al. 1998). Cell‐surface LAMP1 has been associated with more malignant and metastatic cancers, where it promotes drug resistance through increased lysosomal exocytosis (Alessandrini et al. 2017).

In summary, the ability to identify and characterize SEN is crucial for understanding their role in aging and developing targeted interventions. Here, we describe LAMP1 as a cell‐surface‐specific marker of senescence. LAMP1's presence on the cellular membrane is highly increased in human and mouse SEN. In mouse tissue, cells expressing Lamp1 on their surface showed features of senescence and increased with natural aging. Additionally, senescence induction in the lungs of mice, induced by bleomycin (BLM), resulted in an increase in Lamp1^+^ cells. Finally, SEN are eliminated using a LAMP1‐targeting antibody. These findings describe a biomarker that can be leveraged to further understand and target SEN.

## 2 Results

### 2.1 Multi‐Omic Screens Identify LAMP1 Upregulation in Cellular Senescence and Co‐Expression With p21

**FIGURE 1:**
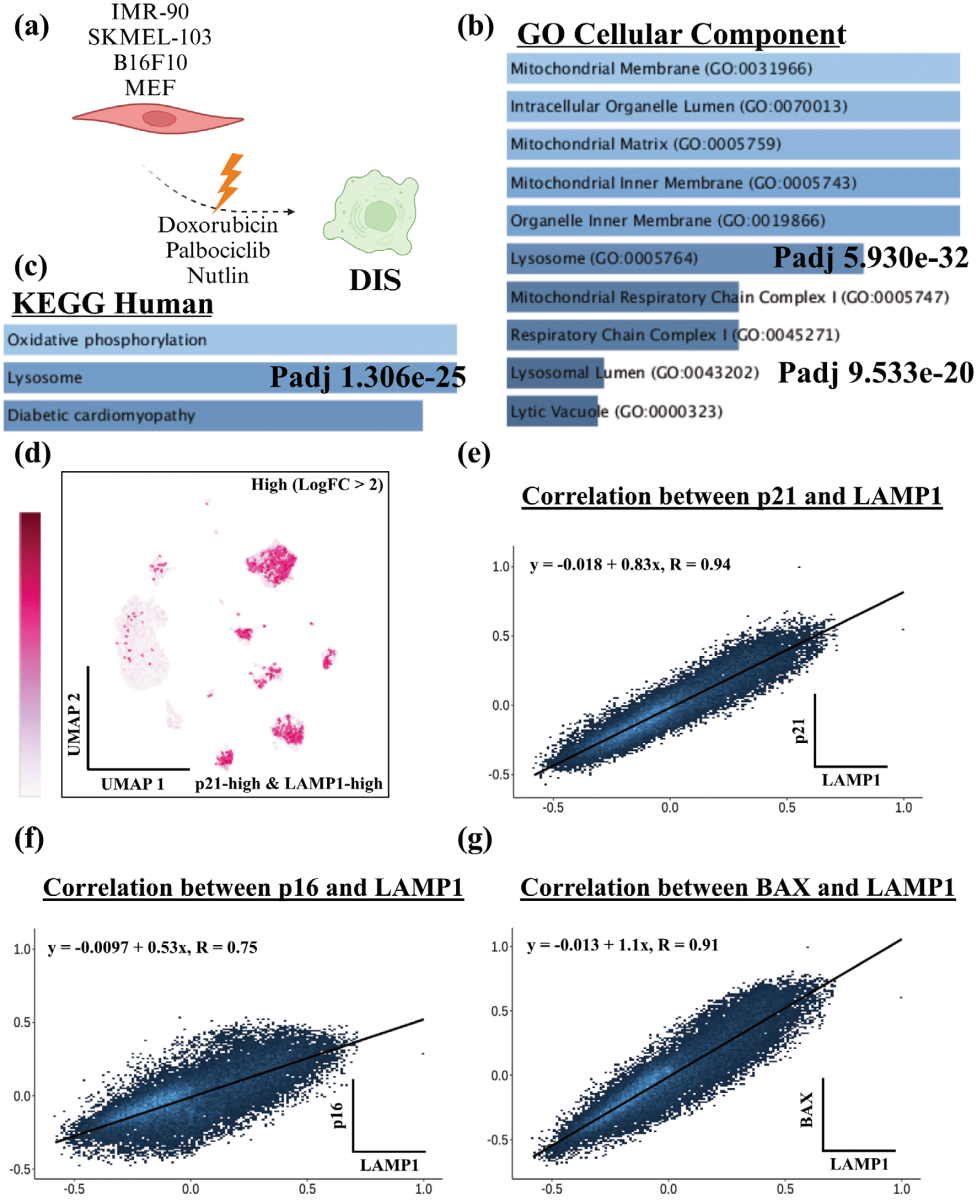
Computational analysis of lysosomal‐associated proteins in senescent cells and old tissue. (a) Schematic of the proteomic screen of the plasma membrane of SEN. IMR‐90, SK‐MEL‐103, B16‐F10, and MEF cell lines were treated with doxorubicin, palbociclib, or nutlin to induce senescence and were collected for proteomic analysis (Marin et al. 2023). Created with BioRender.com. (b, c) Pathway enrichment analysis of proteins that were significantly upregulated on the surface of SEN in at least three conditions (Enrichr). (d) Distinct human muscle cells co‐expressing high (LogFC > 2) LAMP1 and CDKN1A (p21). (e–g) Correlation between LAMP1 expression and senescence‐associated markers (e) p21, (f) p16, and (g) BAX, BCL2‐associated X, apoptosis regulator, in healthy tissue (Correlation AnalyzeR).

We analyzed a publicly available proteomic screen of SEN plasma membrane to identify proteins enriched on the surface of SEN (Marin et al. 2023). This screen contains proteins enriched on SEN of several cell types of origin and modes of senescence induction (Figure 1a). Interestingly, pathway analysis identified the GO terms and KEGG pathways “Lysosome,” “Lysosomal Lumen,” and “Lytic Vacuole” among the top significantly upregulated pathways on the membrane of senescent human fetal lung fibroblasts (IMR‐90s), a melanoma cell line (SK‐MEL‐103), B16‐F10 mouse melanoma cells, as well as mouse embryonic fibroblasts (MEFs) (Figure 1b,c) where damage‐induced senescence (DIS) was induced using chemotherapy drugs (variously doxorubicin, palbociclib, or nutlin). This confirms the fundamental role of lysosomal dysfunction in SEN, which has also been reported elsewhere (Curnock et al. 2023; Rovira et al. 2022).

LAMP1 is essential for lysosomal biogenesis (Rohrer et al. 1996). Analysis of the human muscle aging atlas that consists of almost 300,000 cells isolated from the muscles of young (15–46 years old) and old (74–99 years old) adults revealed that LAMP1 expression was elevated in multiple cell types in aged cells compared to young (Figure S1a,b) (Lai et al. 2024). We further observed a strong correlation of LAMP1 with multiple cell populations with high CDKN1A/p21 expression, a critical mediator of cell cycle arrest in SEN (Figure 1d). SEN are known to be heterogeneous (Admasu et al. 2021; Hernandez‐Segura et al. 2017), so we tested whether this correlation holds across datasets. We interrogated public data from healthy and cancer tissues and observed a strong correlation of LAMP1 expression with other established senescence genes (Miller and Bishop 2021). Not only did LAMP1 have a strong expression correlation with p21 (p21—healthy tissue, *R* = 0.94) (Figure 1e), but its expression was also positively correlated with p16 (Figure 1f) and BAX (BCL2 associated X, an apoptosis regulator) (Figure 1g), which are putative markers of senescence. Moreover, LAMP1 expression strongly correlated with p21 in cancer tissue (*R* = 0.95) and with the surface senescence marker PLAUR (uPAR), which has also been reported as a SEN surface marker and target for senolytic immunotherapy (Amor et al. 2020) (Figure S1c,d).

### 2.2 LAMP1 Is Upregulated on the Cell Membrane of Senescent Cells

**FIGURE 2:**
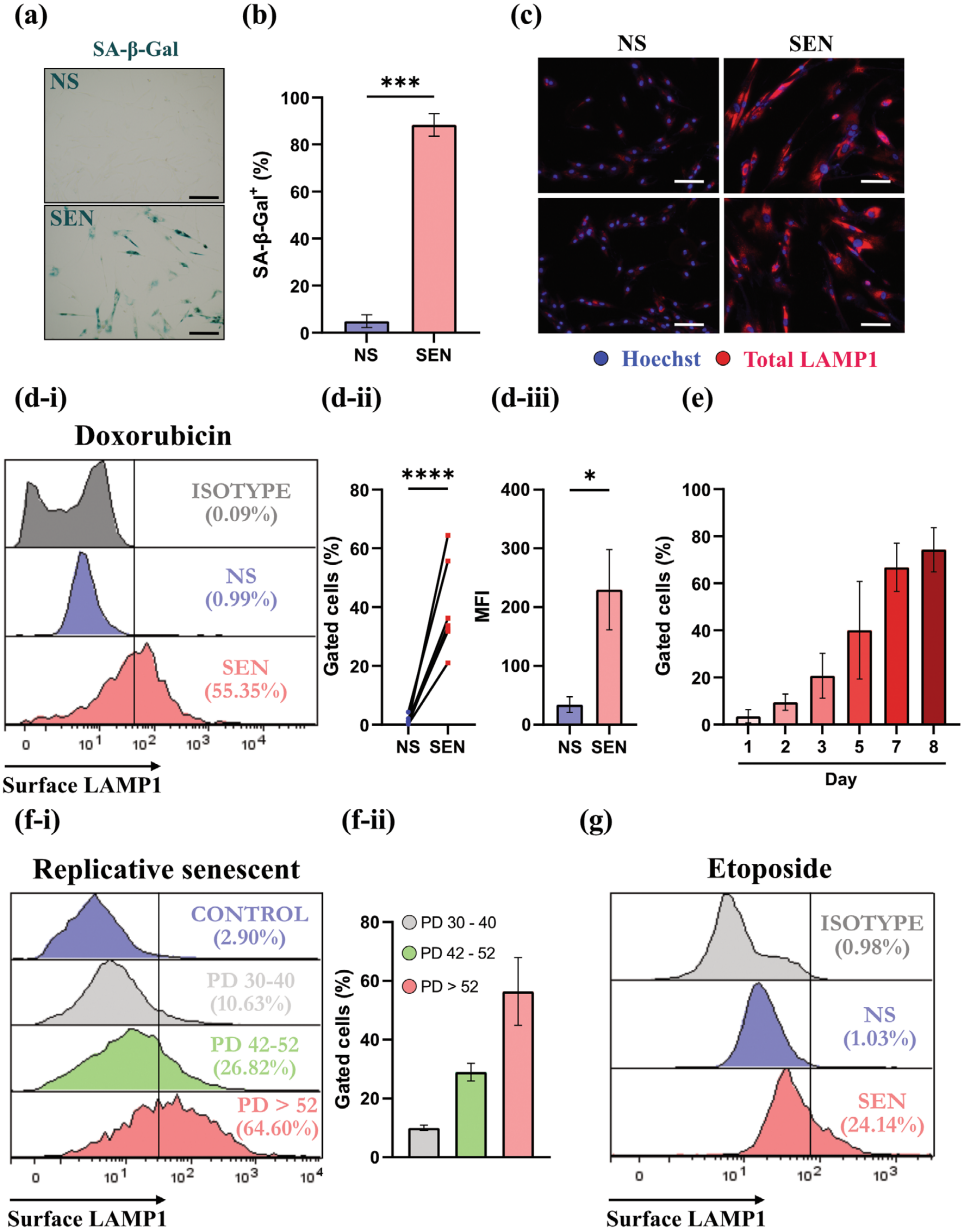
LAMP1 is upregulated on the cellular membrane of senescent cells. (a) SA‐β‐Gal staining in IMR‐90 fibroblasts 9 days after treatment with doxorubicin. Scale bar = 150 μm. (b) Percentage of cells staining positive for SA‐β‐Gal; *n* = 3, unpaired *t*‐test; data represented as mean ± SEM. (c) Representative images of total LAMP1 staining in SEN doxorubicin‐treated fibroblasts and NS controls, *n* = 3. Red, LAMP1. Blue, Hoechst. Scale bar = 150 μm. (d‐i) LAMP1 expression on the surface of SEN and NS measured by flow cytometry. (d‐ii) LAMP1^+^ percentage of analyzed cells; *n* ≥ 3, unpaired *t*‐test. (d‐iii) Mean fluorescence intensity (MFI) of doxorubicin‐treated IMR‐90 fibroblasts stained for LAMP1, *n* ≥ 3, unpaired *t*‐test; data represented as mean ± SEM. (e) LAMP1^+^ cell proportion in senescent IMR‐90 cells at different time points after doxorubicin treatment. *n* = 2; data represented as mean ± SD. (f‐i) LAMP1 cell‐surface staining on IMR‐90 cells of increasing PD numbers; *n* = 2, data represented as gated cells (%). (f‐ii) Quantification of gated cells expressing LAMP1 on their cell surface with progressively higher population doubling number, *n* = 2; data represented as mean ± SD. (g) Representative image (*n* = 3 biological replicates) of etoposide‐treated fibroblasts stained with LAMP1 on the surface of SEN and NS. *p ≤ 0.05; ***p ≤ 0.001; ****p ≤ 0.0001.

To confirm the in silico transcriptomics and proteomics data, we used a well‐characterized doxorubicin model of senescence induction in human fetal lung fibroblasts, IMR‐90. Senescence was established using a 24‐h treatment of doxorubicin (300 nM) followed by 9 days of complete media. After doxorubicin, we observed a significant increase in the percentage of SA‐β‐Gal^+^ IMR‐90 fibroblasts (SEN), compared to untreated non‐senescent cells (NS) (Figure 2a,b). Immunofluorescence confirmed the increased expression of total cytoplasmic and membrane LAMP1 in permeabilized SEN with negligible detection in NS controls (Figure 2c). These results confirmed an increased expression of LAMP1 in the cytoplasm and membrane, as observed in publicly available transcriptomics and proteomics data (Figure 1, Figure S1).

Furthermore, flow cytometric analysis of unfixed and non‐permeabilized SEN and NS IMR90s revealed that only about 1% of NS express LAMP1 on the surface. In comparison, 20% to more than 60% of SEN express stable surface LAMP1 (Figure 2d‐i,d‐ii). Additionally, SEN showed a five‐fold increase in mean fluorescence intensity (MFI) for LAMP1 staining compared to NS controls (Figure 2d‐iii). These observations are consistent with the in silico data, which show increased expression of LAMP1 in SEN and those from older individuals.

Previously, Rovira et al. reported a gradual increase in LAMP1 gene expression in senescent SK‐MEL‐103 melanoma cells (Rovira et al. 2022). To determine the kinetics of LAMP1 expression on the surface of SEN, cells treated with doxorubicin for a varying number of days were analyzed with flow cytometry. We observed a steady increase in the percentage of SEN with surface LAMP1, reaching over 60% by days 7 and 8 and remaining at that level up to day 9 after senescence‐causing insult (Figure 2e).

As the phenotype of SEN may vary depending on the type of initiating damage (Admasu et al. 2021; Hernandez‐Segura et al. 2017), we confirmed the surface expression of LAMP1 in other modes of senescence induction. It is well established that continuous passaging of cells results in replicative senescence (Hayflick and Moorhead 1961). Our results show a progressive increase in surface LAMP1 expression from 10% in cells with PD (population doubling) 30–40 to roughly one quarter in PD 42–52 cells, culminating with more than 60% of replicative senescent cells (PD > 52) exhibiting surface LAMP1, versus negligible levels in controls (Figure 2f‐i,f‐ii). Similarly, etoposide is a well‐known chemotherapy drug routinely used to induce senescence in cultured cells (Georget et al. 2023). Etoposide‐induced senescence was established using a 48‐h treatment with 5 μM etoposide. Cells were maintained for an additional 8 days in cell culture. After etoposide treatment of IMR‐90 cells, we observed an increase in cells positive for SA‐β‐Gal (Figure S2a). 20% of etoposide‐treated IMR‐90 cells expressed LAMP1 on their surface, compared to 1% of NS controls (Figure 2g, Figure S2b,c). Finally, a western blot analysis of the whole cell lysates, lysosomal and membrane fractions (Boominathan et al. 2016; Dixit et al. 2021) of doxorubicin‐treated SEN and NS also confirmed an increase in LAMP1 in the membrane of only SEN. At the same time, an increase in LAMP1 was also detected in the lysosomal fraction compared to samples from NS (Figure S2d).

We observed a low surface expression of LAMP2 in SEN. Only about 10% of SEN were LAMP2^+^, whereas 1% of NS had surface LAMP2 expression (Figure S2e). This data suggests that other lysosomal proteins localize to the cell membrane of SEN.

**FIGURE 3:**
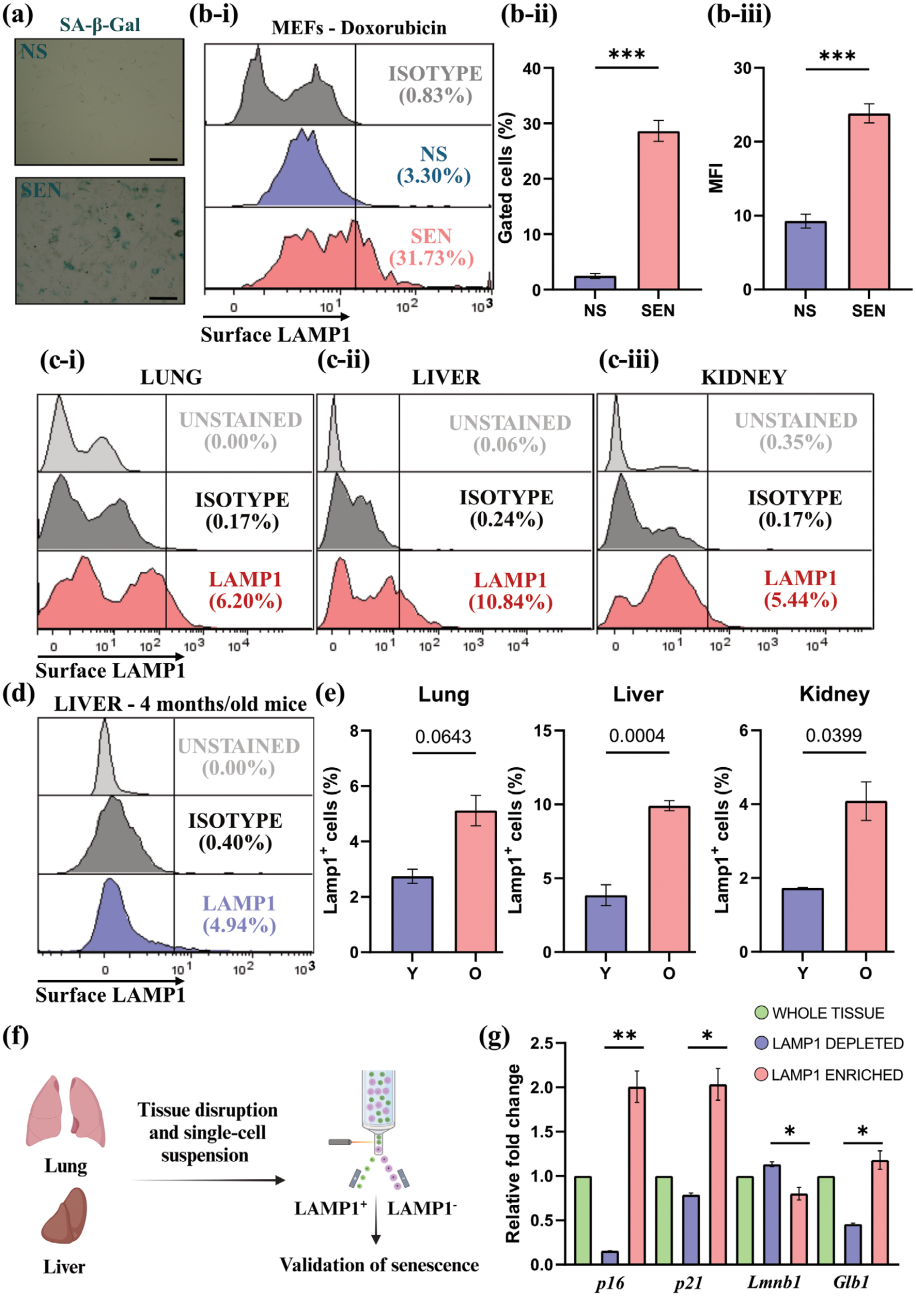
Lamp1^+^ cells express high levels of prototypical senescence markers in mouse tissues. (a) SA‐β‐Gal staining in MEFs 9 days after treatment with doxorubicin. Scale bar = 150 μm. (b‐i) Representative image of Lamp1 expression on the surface of SEN (red) and NS (blue) compared to isotype control (gray). Data is representative of *n* ≥ 3 biological replicates. (b‐ii) Quantification of gated cells expressing Lamp1 on their cell surface. Data is representative of *n* ≥ 3 biological replicates, unpaired *t*‐test; data represented as mean ± SEM. (b‐iii) MFI of SEN and NS stained with Lamp1. Data is representative of *n* ≥ 3 biological replicates, unpaired *t*‐test; data represented as mean ± SEM. (c) Lamp1^+^ population in the lungs (c‐i), liver (c‐ii), and kidneys (c‐iii) of middle‐aged mice (39–63 weeks old). Data is representative of *n* ≥ 2 biological replicates. (d) Representative flow cytometry gating strategy used to quantify Lamp1^+^ cells in the liver of 16‐week‐old mice. Cells were gated based on size, singlets, and viability, and compared to unstained and isotype controls. (e) Percentage of Lamp1^+^ cells in the liver, lungs, and kidneys of middle‐aged mice (39–63‐week‐old) compared to young (16‐week‐old). Data is representative of *n* ≥ 2 biological replicates. (f) Schematic of the experimental approach. Single cells were isolated from mouse tissue and sorted based on their Lamp1 status. RNA was isolated, and RT‐qPCR was used to validate prototypical markers of senescence. Created with BioRender.com. (g) Gene expression of p16, p21, Glb1, and Lmnb1 of Lamp1^−^ and Lamp1^+^ cells. Unsorted single cells were used as controls (green). Data is representative of *n* ≥ 3 biological replicates, unpaired *t*‐test; data represented as mean ± SEM. *Gapdh* and *Actb* were used as housekeeping controls. *p ≤ 0.05; **p ≤ 0.01; ***p ≤ 0.001.

Given that the LAMP1 gene is conserved in mice (mLamp1, or Lamp1), we used doxorubicin to induce senescence in mouse embryonic fibroblasts (MEFs) and NIH/3T3 fibroblasts to confirm our previous observations. MEFs were treated with doxorubicin (150 nM) for 24 h, and senescence was confirmed qualitatively by SA‐β‐Gal after 9 days (Figure 3a). Flow cytometric analysis of unfixed and non‐permeabilized MEFs showed a 10‐fold significant increase in the percentage of SEN MEFs expressing surface Lamp1 compared to NS controls (Figure 3b‐i,b‐ii). The analysis of the MFI in MEFs also confirmed a more than 2‐fold increase in the Lamp1 expression in SEN compared to NS (Figure 3b‐iii). Similarly, senescent NIH/3T3 cells increased the expression of Lamp1 on their surface, with 7% Lamp1^+^ cells in the NS population versus 56% in the SEN population (Figure S3a). This suggests that cell‐surface human and mouse LAMP1 expression is absent or low in NS, but it is markedly higher in SEN.

### 2.3 Lamp1^+^ Cells Express High Levels of Prototypical Senescence Markers in Multiple Mouse Tissues

The age‐dependent increase in senescence burden is well documented (Dimri et al. 1995; Tuttle et al. 2020; Wang et al. 2009). LAMP1 is a widely expressed lysosomal protein (Eskelinen 2006). We set out to determine the surface expression of Lamp1 in the cells from the lung, liver, kidneys, and spleen of middle‐aged mice (39–63‐week‐old animals) in comparison to younger (16‐week‐old) mice. Flow cytometry revealed that younger mice had fewer cells with surface Lamp1 (Figure 3c–e, Figure S3b–d, Table 1). Results show that in the lung, only 2.75% of cells expressed Lamp1 on the surface in the younger mice, compared to 5.12% in older mice (Figure 3c‐i,e, Figure S3d, Table 1). Similarly, in the liver, approximately 4% of cells expressed surface Lamp1 compared to 10% in the older livers (Figure 3c‐ii,e, Figure S3d, Table 1). While in the kidney, the percentage of cells with surface Lamp1 was approximately 2%, compared to 4% in older kidneys (Figure 3c‐iii,e, Figure S3d, Table 1). A similar increase in the proportion of surface Lamp1‐expressing cells was also observed in the spleen (2% in young compared to about 10% in older samples) (Figure S3b,d, Table 1). In summary, at least in the organs studied, approximately half as many Lamp1^+^ SEN were observed in younger mice compared to older mice (Table 1). These results suggest different rates of senescence accumulation in various organs. Overall, our results indicate that surface Lamp1 can be detected in multiple mouse organs, and there is an increase in the proportion of cells that express it with aging (Table 1). Interestingly, these numbers align with previous reports that estimated the senescence burden using other markers in the tissues of middle‐aged, old, or challenged mice (Wang et al. 2024, 2021, 2009).

**TABLE 1:** Percentage of Lamp1^+^ cells observed in different mouse organs.

	Percentage of cell‐surface Lamp1 (%)			
	Young		Old	
	Mean	SD	Mean	SD
Lung	2.75	0.36	5.12	1.45
Liver	3.85	1.23	9.91	0.69
Spleen	2.08	0.89	6.92	4.10
Kidney	1.73	0.01	4.09	1.05

To determine whether the cells positive for surface Lamp1 are indeed senescent, we isolated both Lamp1‐enriched (Lamp1^+^) and Lamp1‐depleted (Lamp1^−^) cell populations from the liver and lungs of middle‐aged mice (Figure 3f). We confirmed high purity in the sorted samples (Figure S3e,f). Consistent with our hypothesis, quantitative real‐time PCR analysis of liver samples revealed that cell‐surface Lamp1^+^ cells express the prototypical markers of senescence, p16 and p21, at higher levels than Lamp1^−^ cells (Figure 3g). Notably, p16 expression in the Lamp1‐depleted population was even lower than in unsorted cells. Presumably, the whole tissue (unsorted population) contains Lamp1^+^ and Lamp1^−^ cells (Figure 3g), leading to the intermediate value. Additionally, Lamp1^+^ cells exhibited features of compromised nuclear integrity, as represented by Lmnb1 downregulation compared to Lamp1^−^ (Figure 3g). Finally, Glb1—the gene encoding lysosomal β‐D‐galactosidase and responsible for SA‐β‐Gal activity—is upregulated in cell‐surface Lamp1^+^ cells. In line with our p16 findings, we observed a slight decrease in the expression of Glb1 in Lamp1^−^ compared to whole tissue or Lamp1^+^ cells (Figure 3g).

Lamp1^+^ cells from the lung had significantly increased expression of p16 but not p21 compared to Lamp1^−^ cells (Figure S3g). In line with previous results, lung Lamp1^+^ cells had lower Lmnb1 expression than Lamp1^−^ cells (Figure S3g). Overall, our results indicate that, at least in the liver and lung, Lamp1‐enriched cells express multiple markers of senescence.

### 2.4 Lamp1^+^ Cells Accumulate in Mice With Fibrosis

Our data so far show that not only do cells undergoing senescence in cell culture express LAMP1 on their surface, but that cell‐surface Lamp1 can be used to enrich cells expressing senescence‐associated genes from various tissues. We next investigated whether this strategy could track senescence burden in pathological states in which SEN are implicated. Idiopathic pulmonary fibrosis (IPF) is a lung disease characterized by progressive and irreversible scarring (fibrosis) of the lung parenchyma (Juarez et al. 2015). The etiology of IPF remains elusive, but its prevalence is strongly associated with advancing age, particularly in those over 50, with a higher incidence in the sixth and seventh decades of life (Selman et al. 2016). Notably, studies demonstrated increased expression of several senescence markers in various types of cells within the lung of IPF experimental models and patients (Parimon et al. 2021).

**FIGURE 4:**
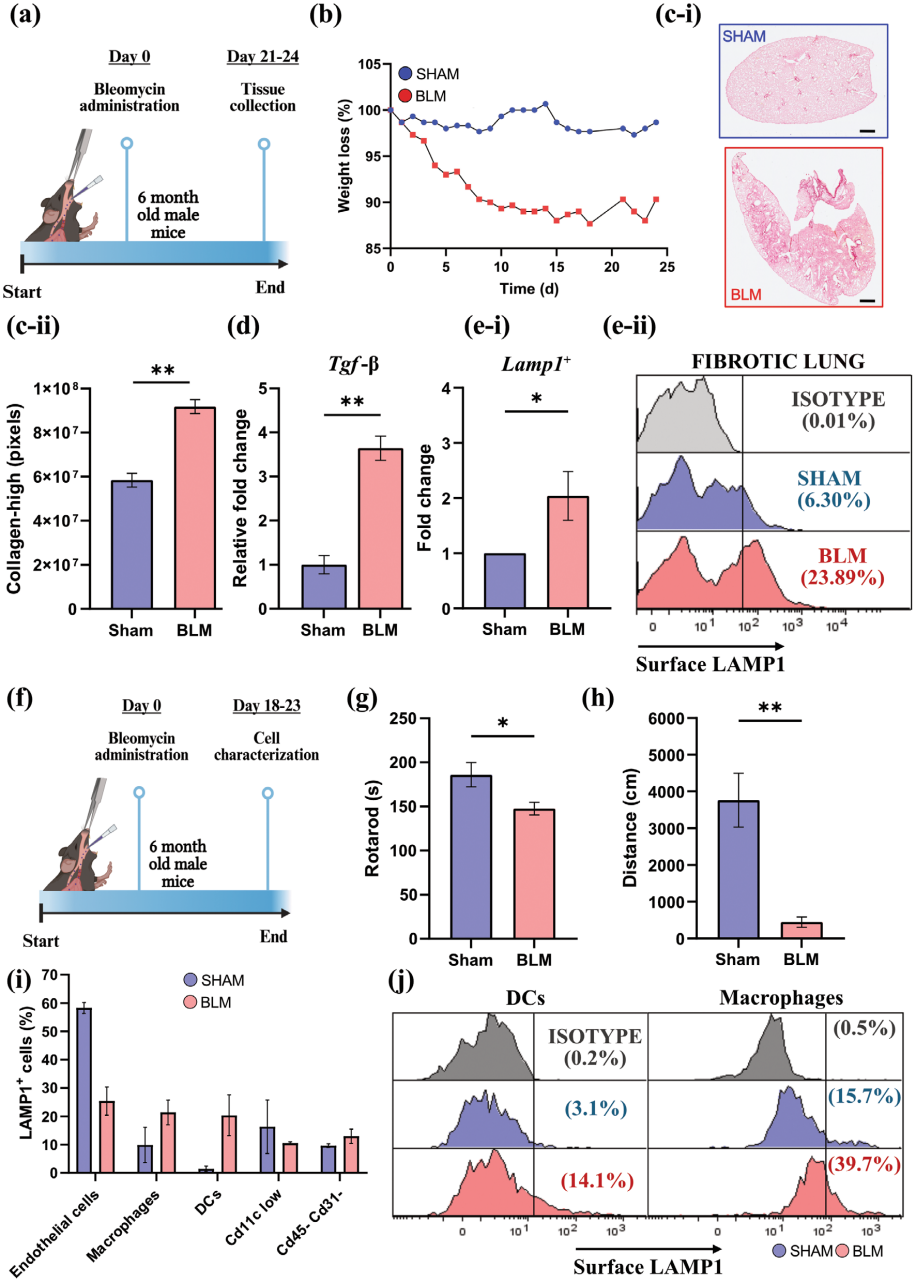
Mice with bleomycin‐induced pulmonary fibrosis have a higher percentage and diversity of Lamp1^+^ cells. (a) Schematic of the experimental design for the induction of fibrosis and senescence in mice. Bleomycin sulfate was delivered on day 0 using the oropharyngeal route of administration. After 24 days, the lungs were collected. Created with BioRender.com. (b) BLM‐induced weight loss. Weight is normalized to the day of BLM instillation (day 0), *n* = 6 (*n* = 3 saline‐treated mice; *n* = 3 BLM‐treated mice). (c‐i) Representative lung sections stained with Picrosirius red. Scale bar = 500 μm. (c‐ii) Quantification of collagen‐high areas (pixels) in controls and BLM‐treated mice (*n* = 3 saline‐treated mice; *n* = 3 BLM‐treated mice); data represented as mean ± SEM, unpaired *t*‐test. (d) Gene expression of *Tgf‐β*. *Hprt* was used as a housekeeping control, data represented as mean ± SEM, unpaired *t*‐test. (e‐i) Fold change increase of Lamp1^+^ cells in the lungs of BLM‐treated mice compared to saline controls. Data represented as mean ± SEM, unpaired *t*‐test. (e‐ii) Representative flow cytometry histogram of Lamp1 expression in saline‐treated controls and BLM‐treated fibrotic lungs. Data is representative of *n* ≥ 3 biological replicates. (f) Schematic of the experimental design for the induction of fibrosis and senescence in mice. Bleomycin sulfate was delivered on day 0 using the oropharyngeal route of administration. After 18–23 days, the lungs were collected to characterize cells expressing Lamp1. Mouse behavior was collected on day 17 (*n* = 7; *n* = 3 saline‐treated mice; *n* = 4 BLM‐treated mice). Created with BioRender.com. (g) Time spent on a rotarod (seconds) (*n* = 7; *n* = 3 saline‐treated mice; *n* = 4 BLM‐treated mice); data represented as mean ± SEM, unpaired *t*‐test. (h) Spontaneous movement was measured for 10 min during open field (*n* = 7; *n* = 3 saline‐treated mice; *n* = 4 BLM‐treated mice); data represented as mean ± SEM, unpaired *t*‐test. (i) Characterization of cells expressing surface Lamp1 in the BLM‐treated fibrotic pooled lungs and the saline‐treated mice pooled lungs. Data are representative of *n* = 3 saline‐treated mice and *n* = 4 BLM‐treated mice. (j) Representative flow cytometry histogram of Lamp1 expression in saline‐treated controls and BLM‐treated fibrotic lungs. Live, Cd45^+^, Cd11c^+^, and SiglecF^−^ cells were considered dendritic cells (DCs) (left panel). Live, Cd45^+^, Cd11c^+^, and SiglecF^+^ cells were considered macrophages (right panel). *p ≤ 0.05; **p ≤ 0.01.

It is well established that the genotoxic agent BLM induces pulmonary fibrosis in humans (Mohammed et al. 2024) and in the mouse lung (Schafer et al. 2017). In this study, we explored whether cell‐surface LAMP1 may serve as a biomarker of SEN in mice after IPF induction. As illustrated in Figure 4a, BLM or saline was administered to mice via the oropharyngeal route. Following BLM instillation, the weight of the treated mice decreased, suggesting successful IPF induction (Figure 4b). Previously, extensive fibrotic lesions have been reported in the lungs of mice 20–28 days post‐BLM challenge (Schafer et al. 2017). The Picrosirius red stain is one of the widely used histochemical techniques for staining collagen and detecting changes in matrix organization characteristic of fibrosis (Vogel et al. 2015). Our data confirmed significantly increased collagen deposition in the lungs of BLM‐treated mice compared to sham‐treated mice (Figure 4c‐i,c‐ii). An increase in TGF‐β primarily drives collagen deposition in the lungs of patients with IPF (Gimenez et al. 2017). Real‐time qPCR analysis of the lung tissue confirmed a three‐fold increase in the expression of *Tgf‐β* in BLM‐treated compared to sham‐treated mice (Figure 4d). Additionally, real‐time qPCR analysis also confirmed an increase in the expression of p21 and p16 in lung tissue from BLM‐treated mice compared to that of sham‐treated mice (Figure S4a).

As we previously found that cell‐surface Lamp1 expression is associated with a senescent phenotype, we next set out to analyze single cells prepared from the lungs of sham‐ and BLM‐treated mice. Viable cells from whole‐lung single‐cell suspensions were analyzed for Lamp1 surface expression (Figure S4b). Results show a 1.5‐ to 3‐fold increase in the number of Lamp1^+^ cells after the BLM challenge relative to sham treatment (Figure 4e‐i,e‐ii). All BLM‐treated mice showed an increase in Lamp1^+^ cells, but there was mouse‐to‐mouse variability in the degree of increase (Figure 4e‐i). Our data indicate a greater number of Lamp1^+^ cells in mice with an increased senescence burden following BLM treatment.

Previous reports found an increase in the senescence phenotype in different cell types collected from IPF lungs, including endothelial cells (ECs), epithelial cells, AT2 cells, basal cells, fibroblasts, mesenchymal progenitor cells, and innate and adaptive immune cells (Parimon et al. 2021; Schafer et al. 2017). We wondered if an increase in Lamp1 on the cell surface also occurs in multiple cell types in the fibrotic mouse lung. To this end, we repeated the BLM instillation to induce lung fibrosis (Figure 4f). Running time on a rotarod and spontaneous activity were recorded as a measure of general condition deterioration after the treatment, indicative of fibrosis development. Results from the rotarod test reveal a significant decline in the time mice stayed on the rotating rod following BLM instillation (Figure 4g). The open field test evaluates general activity levels, gross locomotor activity, and exploration habits in mice. Animals were placed into an open arena and allowed to freely explore while data was gathered using an electronic tracking system (Noldus Ethovision). We confirmed a decline in spontaneous activity and the total distance moved in the BLM‐treated mice compared to sham control mice (Figure 4h). To determine which lung cell types express Lamp1 on the surface, we designed a multicolor flow cytometry panel that identifies several cell lineages. Our results show that the majority of Lamp1^+^ cells in the lungs of sham‐treated mice were ECs (as defined by Cd45^−^, Cd31^+^) (Figure 4i). Small populations of Cd45^+^, Cd11c‐high, Siglec‐high macrophages, as well as Cd11c‐low and undefined Cd45^−^ cells also expressed surface Lamp1 in the sham mice. By contrast, we observed multiple distinct cell types expressing surface Lamp1 after BLM instillation. Most of these cells were ECs, macrophages, and dendritic cells (defined as Cd45^+^, Cd11c‐high, Siglec‐low) (Figure 4i,j). In contrast, only a small percentage of T cells (Cd45^+^, Cd11c‐low, Cd3e^+^) in the BLM‐treated mice expressed surface Lamp1 (Figure S4c).

**FIGURE 5:**
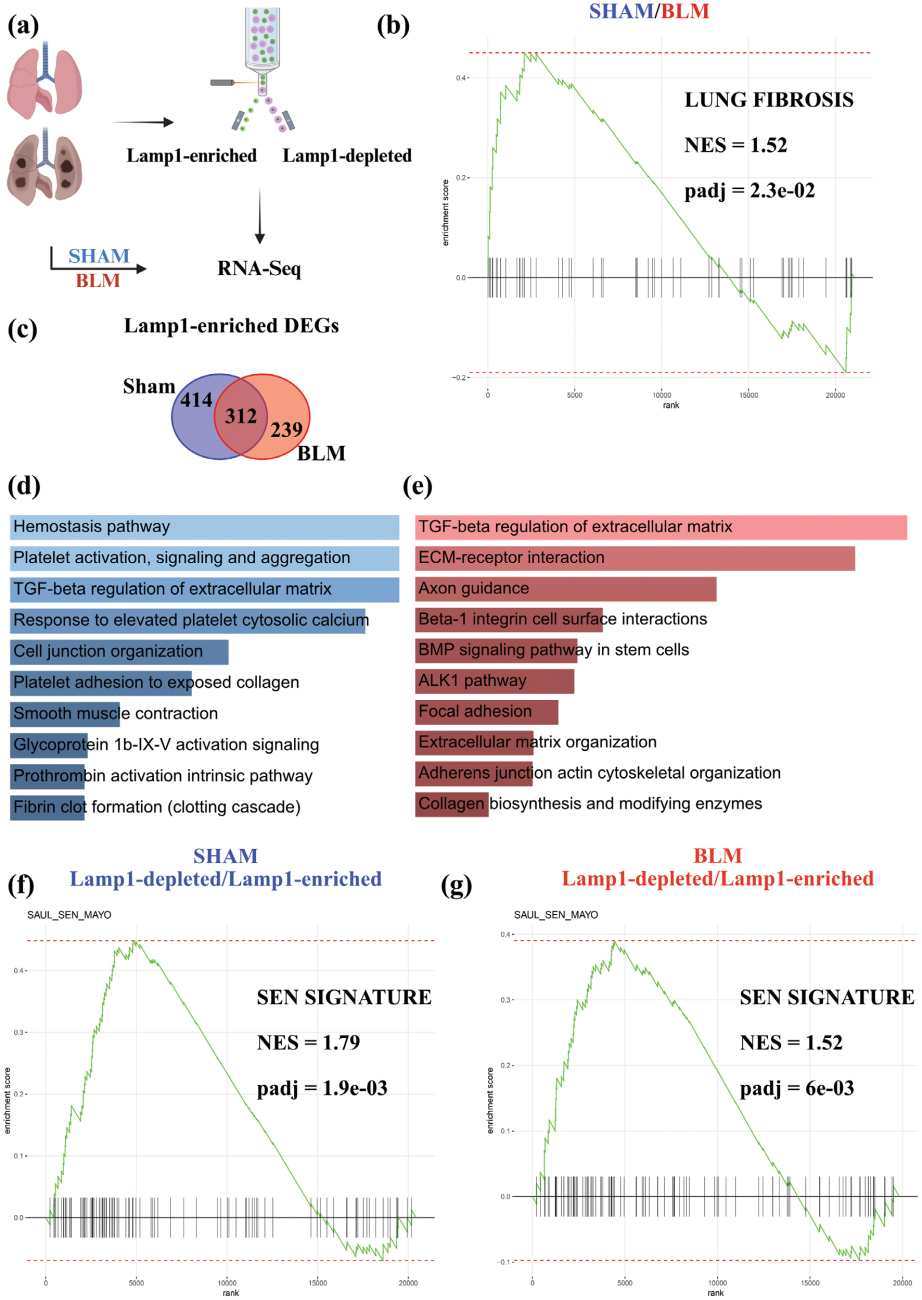
Lung Lamp1‐enriched cells have a senescent gene signature. (a) Schematic of the experimental design for the induction of fibrosis and senescence in mice. Bleomycin sulfate was delivered on day 0 using the oropharyngeal route of administration. After 21 days, the lungs were harvested, and whole‐tissue single cells were collected for sequencing. Additionally, cells were sorted based on surface Lamp1 expression and collected for sequencing. Created with BioRender.com. (b) GSEA of sham‐treated mice compared to BLM‐treated mice for the pathway “WP_LUNG_FIBROSIS.” (c) Upregulated genes in the Lamp1‐enriched samples in sham‐ (blue) and BLM‐treated (red) mice. Light pink represents genes upregulated in both sham and BLM when comparing Lamp1‐enriched versus Lamp1‐depleted cells. (d) Upregulated pathways in Lamp1‐enriched cells in the sham‐treated mice (BioPlanet 2019). All pathways had a *p*adj < 0.05. Pathways on top had lower *p*adj values. (e) Upregulated pathways in Lamp1‐enriched cells in the BLM‐treated mice (BioPlanet 2019). All pathways had a *p*adj < 0.05. Pathways on top had lower *p*adj values. (f, g) GSEA of sham‐treated mice and BLM‐treated mice for the pathway “SAUL_SEN_MAYO.” Lamp1‐depleted cells were compared to Lamp1‐enriched cells for (f) sham‐treated mice and (g) BLM‐treated mice.

To comprehensively understand the phenotype of cell‐surface Lamp1 cells, we performed Bulk RNA‐Sequencing (RNA‐Seq.) of Lamp1‐enriched, Lamp1‐depleted, and unsorted cell suspensions from the whole lung in sham‐treated and BLM‐treated mice 21 days after BLM instillation (Figure 5a). We observed a significant increase in fibrotic genes in unsorted lung cells from BLM‐treated mice compared to those from sham‐treated mice (Figure 5b). We compared the upregulated genes in Lamp1‐enriched cells in sham‐treated mice versus those in Lamp1‐enriched cells in the BLM‐treated mice. We observed an overlap of 312 genes between both datasets (Figure 5c). Lamp1‐enriched cells from sham‐treated mice had different pathways upregulated compared to Lamp1‐enriched cells from BLM‐treated mice (Figure 5d,e). Notably, pathways related to pulmonary fibrosis, such as TGF‐β regulation, extracellular matrix organization, or collagen biosynthesis, were preferentially upregulated in Lamp1‐enriched cells from the BLM‐treated mice (Figure 5e). In line with our hypothesis, Lamp1‐enriched cells showed a clear senescence signature compared to Lamp1‐depleted cells according to the SenMayo gene panel, both in sham‐treated mice and BLM‐treated mice (Saul et al. 2022) (Figure 5f,g, Figure S5a, Appendix S1–, S3).

### 2.5 A LAMP1‐Targeting Antibody‐Drug Conjugate (ADC) Targets Senescent Cells

**FIGURE 6:**
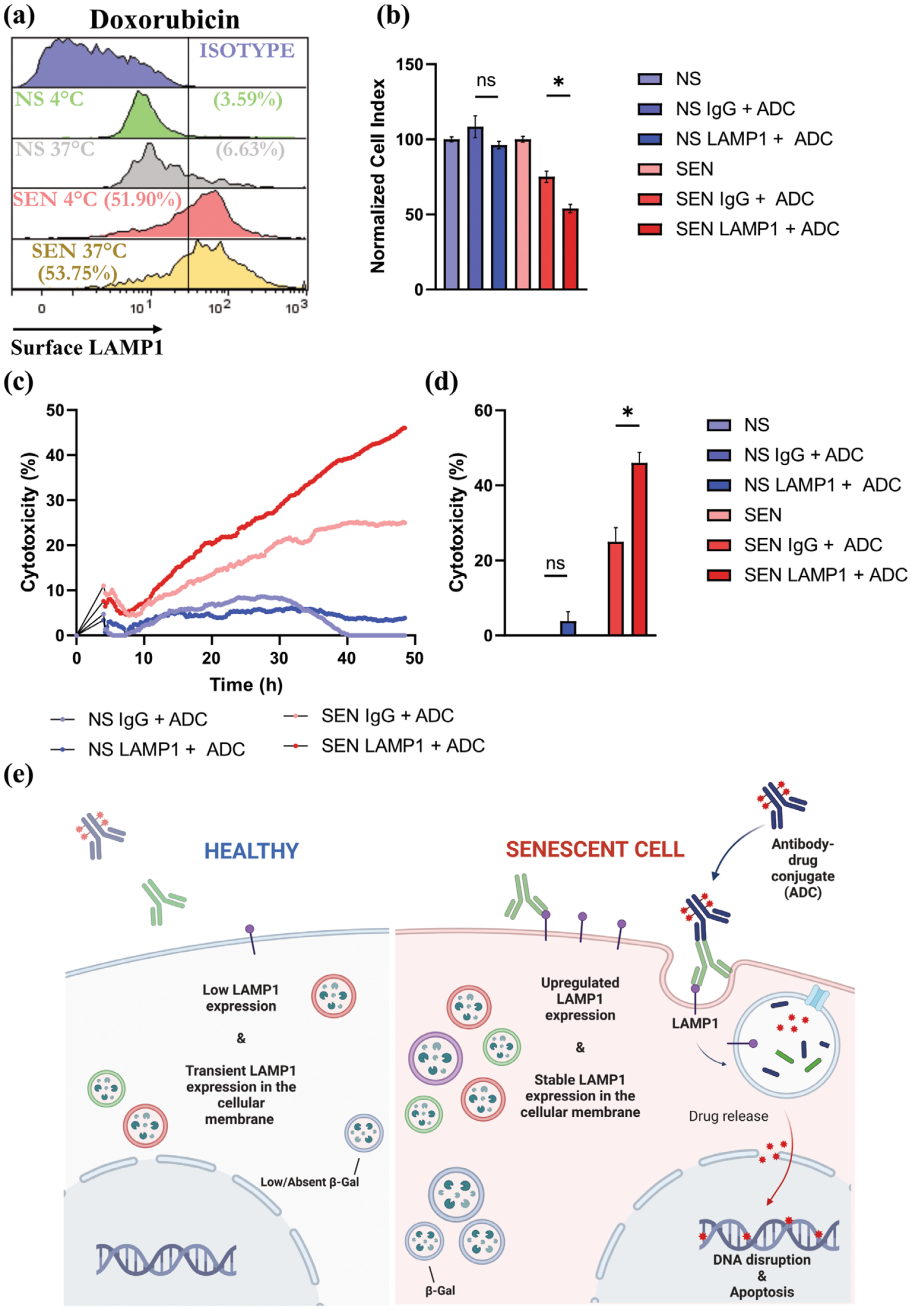
A LAMP1‐ADC kills senescent cells. (a) LAMP1 expression on the surface of SEN and NS IMR‐90 at 4°C and 37°C. Data is representative of *n* ≥ 3 biological replicates. (b) Normalized cell index in SEN and NS IMR‐90 after 48 h of labeling with LAMP1 plus ADC, IgG control plus ADC, or nothing. Data is representative of *n* ≥ 3 biological replicates; mean ± SEM, unpaired *t*‐test. (c) Real‐time kinetics of ADC killing NS and SEN IMR‐90 as measured by the xCELLigence RTCA‐MP platform every 15 min. Untreated NS and SEN IMR‐90 were used as controls (0% death). Data is representative of *n* ≥ 3 biological replicates. (d) Cytotoxicity in SEN and NS IMR‐90 after 48 h of labeling with LAMP1 plus ADC, IgG control plus ADC, or nothing. Data is representative of *n* ≥ 3 biological replicates; mean ± SEM, unpaired *t*‐test. (e) ADC approach to target SEN while sparing healthy NS schematic. Created with BioRender.com. *p ≤ 0.05.

Finally, we wanted to assess whether SEN cell‐surface LAMP1 can be exploited to target and clear SEN. First, to test LAMP1 surface expression in physiological conditions, we performed LAMP1 labeling for flow cytometry at both 4°C and 37°C. We observed that LAMP1 remained stable and selectively elevated on the surface of SEN in both conditions (Figure 6a). We then utilized an ADC approach to test if cell‐surface LAMP1 can be targeted to selectively deliver a cytotoxic payload to SEN. IMR‐90 SEN and NS controls were incubated with either a human anti‐LAMP1 antibody or an IgG control antibody. Following incubation, a secondary ADC antibody targeting the primary anti‐LAMP1 antibody and conjugated with a cytotoxic duocarmycin payload was added to the wells. To assess cell viability, we used the xCELLigence RTCA‐MP (Agilent, USA), which tracks real‐time cell viability by measuring the cellular impedance of live cells (Ke et al. 2011). Changes in impedance are expressed as a cell index (CI) value, which derives from relative impedance changes corresponding to cellular coverage of the electrode sensors, normalized to baseline impedance values with medium only. Percent cytotoxicity was determined based on the normalized CI value of cells treated with the antibody‐ADC sequence relative to control cells untreated with either. Real‐time killing was measured every 15 min for 48 h. Results show a significant decline in the normalized CI in SEN treated with the two‐part anti‐LAMP1 ADC strategy compared to the cells treated with the IgG control antibody plus ADC. In contrast, this treatment did not affect the viability of the NS control (Figure 6b). Our results further show an increase in percent cytotoxicity in SEN following treatment with the two‐part ADC within 10 h and a steady increase in the cytotoxicity to around 50% by the 48 h mark. We did not observe any increase in cytotoxicity in NS treated with this approach within 48 h (Figure 6b–d). We conclude that SEN can be targeted using an anti‐LAMP1 ADC treatment (Figure 6e).

## 3 Discussion

Identifying SEN in living tissue based on the activity of SA‐β‐Gal has been critical for understanding the biology of aging and the role of cellular senescence in physiology (Dimri et al. 1995). Since then, several other markers have been used to identify SEN, including increased expression of the cell cycle checkpoint inhibitors p16 and p21, SASP factors such as IL‐6, IL‐8, and MMP9, and release of the DAMP signaling molecule HMGB1 into the extracellular milieu (Hernandez‐Segura et al. 2018). Use of these markers has enabled the discovery of the role of SEN in the biological aging process and diseases of aging. It has identified SEN as targets for the treatment of numerous diseases associated with aging and for degenerative aging in general (Kirkland and Tchkonia 2017). However, no known “unique” senescence markers exist, and most existing markers are useful in cell culture models but have limited application in the identification of senescence in tissues or require transgenic models that cannot be translated to human medicine. The lack of specific and broadly applicable markers of SEN in tissues and living organisms has led to guidelines called “minimum information for cellular senescence experimentation in vivo” (MICSE) that recommend measuring at least two or more markers for greater confidence (Ogrodnik et al. 2024).

Here, we build upon prior findings that lysosomal dysfunction is characteristic of cellular senescence and is often associated with aging and age‐related diseases (Curnock et al. 2023; Rovira et al. 2022). Analysis of previously published single‐cell RNA‐Sequencing (scRNA‐Seq.) data shows a strong correlation of expression of the lysosomal membrane protein LAMP1 with p21. This was further confirmed by studying the correlation of LAMP1 with several senescence phenotypes in healthy and cancer tissue samples, suggesting its involvement in the senescence cell fate. Increased expression of lysosomal proteins during senescence has been previously reported (Curnock et al. 2023; Deng et al. 2024; Kang et al. 2017; Marin et al. 2023; Rovira et al. 2022). In agreement with these studies, we observed an increase in the expression of total LAMP1 in SEN by immunofluorescence assay. While no previous studies have focused on LAMP1 as a selective and sensitive marker of senescence, it has been identified among over‐represented cell‐surface proteins in several prior reports (Deng et al. 2024; Marin et al. 2023). A surfaceome mass spectrometry screen of NHLFs, MEFs, primary mouse astrocytes, and HUVECs induced to senesce using several methods (genotoxic stress, oxidative stress, or proteasome‐induced stress) revealed that LAMP1 was among the top 27 differentially expressed proteins (DEPs) in the membrane of SEN (Deng et al. 2024). An increase in the LAMP1 expression has also been previously reported on the surface of SEN from the human melanoma line SK‐MEL‐103 (Rovira et al. 2022) and in the membrane fraction of etoposide‐treated SEN (Kang et al. 2017).

In this study, we observed about 10% of cells that were Lamp1^+^ in the liver of 39–63 weeks old mice, which is similar to the 8.4% ± 1.7% of hepatocytes deemed senescent in mice of a similar age via γH2AX foci (Wang et al. 2009) and expectedly lower than the 15% of hepatocytes deemed senescent in 24‐month‐old mice using TAF (Ogrodnik et al. 2017). The quantitative real‐time PCR analysis of Lamp1^+^ cells further confirmed that the expression of several other senescence markers (p16, p21, and Glb1) was elevated in these cells compared to Lamp1^−^ cells, with the unsorted mixed population being intermediate between the two. The entire proteome of senescent Lamp1^+^ cells in humans and mice remains uncharacterized.

Our results show that around 6% of cells in the lung of middle‐aged mice express Lamp1 on the surface, which not only had significantly higher *p16* but also a lower *Lmnb1* expression compared to the cells without surface Lamp1. Similarly, Wang et al. reported 6.7% SEN based on TAF in the lungs of 12‐month‐old mice (Wang et al. 2009), and Biran et al. reported about 6%–7% SA‐β‐Gal^+^ cells in the lungs of older (24‐month‐old) mice (Biran et al. 2017). Additionally, our data show little senescence burden in young mice, which increases with age. An increase in senescence burden is a characteristic of several pathologies, including inflammatory lung diseases like IPF and COPD (Schafer et al. 2017). An increase in the expression of SA‐β‐Gal, p16, or p21 in the lungs of mice treated with BLM is also well documented (Biran et al. 2017; Schafer et al. 2017). Consistent with these findings, our data from the BLM‐treated mouse model of IPF also confirmed a significant increase in the proportion of cells in the lung expressing Lamp1 on the surface, with a high degree of mouse‐to‐mouse variability (9%–24%).

Lamp1 is also known to be transiently expressed on the surface of many cell types, most notably immune cells such as activated natural killer (NK) and T cells (Cohnen et al. 2013). In the BLM‐treated mice, cells expressing surface Lamp1 were predominantly macrophages and ECs, with smaller numbers of other types of cells, like dendritic cells. In sham‐treated mice, most cells that expressed Lamp1 on their surface were ECs. Others have previously shown a senescent phenotype in different types of cells in the IPF lungs (Parimon et al. 2021). In contrast, we did not observe a change in the proportion of fibroblasts or epithelial cells in sham versus BLM‐treated mice lungs based on surface Lamp1 expression, even though AT2 cells in the lung are often associated with increased senescence burden (Yao et al. 2021). Mesenchymal stem cells (MSCs) express a significant amount of the protein LAMP1 on their cell surface when undergoing differentiation into adipocytes. However, in the lung, this is a rare population (Xu et al. 2020). Interestingly, a recent study using genetic tools to trace the functional role of p16‐expressing cells reported that senescent macrophages and ECs represent distinct cell populations with different fates and functions during liver fibrosis and repair, as removal of p16‐expressing macrophages significantly reduced tissue damage, whereas p16‐ECs were involved in tissue repair and regeneration (Zhao et al. 2024). In line with this, we observed that most Lamp1^+^ cells in the sham‐treated mice were ECs. After BLM challenge, we observed an increase in macrophage‐expressing cell‐surface Lamp1. This finding invites more investigations into the role of senescence in different cell types in the pathology of IPF. Whether this phenomenon also exists in other age‐related pathologies needs to be studied in the future. Additionally, it presents an intriguing possibility to test whether the selective delivery of senolytic interventions to specific cell types (such as senescent macrophages instead of senescent ECs) may further refine the efficacy of senolytic interventions in resolving IPF. Our data showed that different cell types have different predispositions to acquire a senescent phenotype. Heterogeneity in senescence based on the cell type warrants further investigation. The RNA‐Seq. analysis of the Lamp1‐enriched populations in sham and BLM mice lung tissue revealed enrichment of several senescence‐related genes in both groups when compared to the SenMayo gene set derived from transcriptomic profiling of senescence markers in Mayo Clinic research datasets, further confirming the senescence phenotype in the Lamp1 enriched populations.

Recent studies have investigated immune surveillance of SEN in aging and disease (Burton and Stolzing 2018; Pereira et al. 2019; Sagiv et al. 2016; Zhang et al. 2022). These efforts have led to the development of immune‐based therapeutic interventions for treating age‐related diseases (Arora et al. 2021; Yang et al. 2023). The discovery and validation of several SEN surface markers, including ApoD, DPP4, and PLAUR (uPAR), open the door to clearing SEN by targeting these antigens using engineered lymphocytes (Amor et al. 2020; Burton and Stolzing 2018; Kim et al. 2017; Poblocka et al. 2021; Rossi and Abdelmohsen 2021; Takaya et al. 2023; Yang et al. 2023). uPAR‐specific CAR‐T cells efficiently ablate SEN in vitro and in vivo, extend the survival of mice with lung adenocarcinoma treated with senescence‐inducing chemotherapy, and restore tissue homeostasis in mice in which liver fibrosis is induced chemically or by diet (Amor et al. 2020). Moreover, treatment with anti‐uPAR CAR‐T cells ameliorates age‐associated metabolic dysfunction, as evidenced by improvements in glucose tolerance, in aged mice and those on a high‐fat diet (Amor et al. 2024). However, CAR‐T therapeutic interventions are expensive and not free from potential adverse effects (Chohan et al. 2023).

Here, we performed proof‐of‐principle experiments to test the therapeutic targeting of SEN based on the expression of surface LAMP1 using a two‐part ADC. We measured cytotoxicity using the xCELLigence RTCA‐MP platform, which enables continuous monitoring of cell viability, unlike other endpoint‐only methods. Others have tried similar approaches and time points to evaluate cytotoxicity with other surface markers, including ApoD (Takaya et al. 2023) and DPP4 (Kim et al. 2017). The results from cell culture data were promising and should be tested in vivo in the future.

Our results demonstrated that several types of SEN express LAMP1 on the surface. However, the heterogeneity of the senescence phenotype requires further investigation. While initially thought to be a uniform phenotype, recent research has revealed significant variability in the senescence phenotype, driven by factors such as cell type of origin, cell cycle, and the nature of the inducing stimulus (Admasu et al. 2021; Hernandez‐Segura et al. 2017). Advances in scRNA‐Seq. and other “omic” technologies enable the detailed characterization of SEN heterogeneity at the single‐cell level, revealing diverse characteristics and behaviors of SEN within a population and providing insights into the underlying molecular mechanisms (Cohn et al. 2023). Future research will provide a deeper understanding of the diversity of Lamp1^+^ cells in the lung and other organs, shedding light on their distinct roles in driving aging and age‐related diseases. Additionally, unbiased proteomics will elucidate the proteome of Lamp1^+^ SEN and their SASP.

Another interesting question that remains to be answered is the mechanism by which LAMP1 is presented on the surface of SEN. It has been hypothesized that proteins from the lysosome and other phospholipid‐rich bilayer organelles could be stably present in the plasma membrane as a mechanism to repair or support the damaged cell surface (Corrotte et al. 2015; Reddy et al. 2001; Sarafian et al. 1998). When the cellular surface is damaged, lysosomes may fuse with the plasma membrane to stabilize the lipid bilayer. This hypothesis is supported by a report from Reddy et al. who described a Ca^2+^‐regulated exocytosis mechanism that mediates plasma membrane damage repair (Corrotte et al. 2015; Idone et al. 2008). We have also recently demonstrated an increase in lysosomal exocytosis in SEN, which is regulated by the PIKfyve kinase. Inhibition of this kinase leads to senolytic activity in cell culture and a mouse model of IPF (Barkovskaya et al. 2025). Our results also show an increase in the surface expression of LAMP2 in SEN (Figure S2e). However, the expression was lower compared to LAMP1 in SEN. Whether other lysosomal membrane proteins could be used to target SEN warrants further investigation.

LAMP1 is typically localized on lysosomal membranes, but it can also be transiently displayed on the cell surface under certain conditions, which is of particular interest in immunology and cancer research due to its role in cell signaling, migration, and immune modulation (Krzewski et al. 2013). For instance, in immune cells, such as T cells and NK cells, LAMP1 is commonly translocated to the cell surface as a marker of degranulation or cellular activation. This occurs when cytotoxic granules (lysosome‐like organelles) fuse with the plasma membrane, resulting in the display of LAMP1 and other lysosomal proteins on the cell surface. LAMP1 can also reach the cell surface via vesicular transport from the trans‐Golgi network (TGN) or endosomes. After being synthesized and glycosylated in the endoplasmic reticulum (ER) and Golgi, LAMP1 is directed to lysosomes through clathrin‐coated vesicles with mannose‐6‐phosphate receptors or other sorting receptors. However, a small fraction of LAMP1 can bypass the lysosomes and be trafficked directly to the plasma membrane through secretory vesicles, particularly under conditions that impair normal lysosomal trafficking. While these are examples of transient processes by which LAMP1 may be briefly displayed on the cell surface under homeostatic conditions, many tumor cells show a persistent increase in surface expression of LAMP1, which has been linked to their ability to evade immune detection and metastasize. The expression can be upregulated under cellular stress, such as hypoxia or nutrient deprivation, which induces autophagy and lysosomal biogenesis. These conditions increase lysosomal exocytosis and result in greater LAMP1 display on the surface (Agarwal et al. 2015). This aberrant increase in cell‐surface LAMP1 expression is more reminiscent of the stable presence of LAMP1 on the cell surface of SEN that we report here and awaits further investigation.

As stated earlier, a recent study from our laboratory has shown that the process responsible for presenting LAMP1 on the surface of SEN is a vital pro‐survival mechanism, and its inhibition has been reported to be senolytic (Barkovskaya et al. 2025). However, whether the surface expression of LAMP1 is merely the consequence of this process is not clear. One way to investigate this would be to manipulate the expression of LAMP1 in SEN by knockdown. Interestingly, Andrejewski et al. have reported that LAMP1 knockout mice are not only viable and fertile, but their lysosomal properties are not affected, presumably because of compensatory upregulation of LAMP2 (Andrejewski et al. 1999). It would be intriguing to characterize senescence in these mice.

In conclusion, our results show that LAMP1 is upregulated on the surface of SEN compared to NS controls. LAMP1 is upregulated on the membrane in human cells that have been induced to senesce through various mechanisms. Similarly, Lamp1 is upregulated in multiple senescent mouse cell lines. We demonstrate that SEN can be distinguished from tissues based on the expression of Lamp1 on the cell surface. Finally, we confirmed our findings in vivo using a fibrosis mouse model, where we found a greater abundance of Lamp1^+^ cells. In summary, we propose lysosomal‐associated membrane proteins, particularly LAMP1, as selective markers of cellular senescence in vitro and in vivo.

## 4 Materials and Methods

### 4.1 Cell Culture and Senescence Induction

IMR‐90 (human lung fibroblasts) and MEFs (mouse embryonic fibroblasts) were cultured in DMEM medium (Corning) supplemented with 10% fetal bovine serum (Sigma) and Penicillin–Streptomycin (Corning) in a humidified atmosphere with 5% CO_2_, 3% O_2_ at 37°C. Cells were routinely checked for mycoplasma.

To induce senescence in vitro, IMR‐90 cells were treated with 300 nM doxorubicin (Tocris) for 24 h, then kept in regular culture medium for 9 days before cell collection. Senescence establishment was evaluated 10 days after doxorubicin treatment. Alternatively, IMR‐90 cells were treated with 5 μM etoposide (ApexBio) for 48 h and maintained in culture for 8 days. MEFs received 150 nM of doxorubicin for 24 h and were considered senescent after 9 days post‐challenge. To achieve replicative senescence, IMR‐90 cells were passaged continuously until the exhaustion of replication. Replicative senescence was confirmed by SA‐β‐Gal assay. Assays with SEN were done 10 days after drug‐induced senescence.

### 4.2 Senescence‐Associated β‐Galactosidase

SA‐β‐Gal staining was performed using a Senescence Detection Kit (Abcam) following the manufacturer's instructions. Shortly, cells were fixed using a fixative solution, washed, and stained with a staining solution containing 20 mg/mL X‐Gal. Cells were incubated for 24 h, without CO_2_, and imaged.

### 4.3 Animals

All animal protocols were reviewed and approved by the IACUC. Male C57BL/6J mice used for modeling idiopathic pulmonary fibrosis were purchased from the Jackson Laboratory. All mice were given food and water ad libitum. To induce pulmonary fibrosis, 3 or 3.5–4 mg/kg of Bleomycin sulfate (MedChem Express) was administered using the oropharyngeal administration route. Mice were weighed daily and assessed for well‐being by a trained specialist. Mice were given additional food and gels to mitigate BLM‐induced weight loss. Lungs were collected 18–24 days after BLM instillation. For validation of fibrosis, the left lobe of the lung was collected for histology, and the right lobes were immediately flash frozen and used for RT‐qPCR. For flow cytometry, whole lungs or left and right lobes were dissociated and stained as described below.

### 4.4 Histology

Lungs were harvested and fixed in 10% neutral buffered formalin for 24 h before being transferred to 70% ethanol prior to paraffin embedding. 5 μm paraffin‐embedded sections were deparaffinized, rehydrated, and treated with 0.2% phosphomolybdic acid aqueous solution for 5 min. Sections were then stained with Picrosirius red prepared in 0.1% saturated aqueous solution of picric acid for 2 h at room temperature. The sections were washed with 0.015 N hydrochloric acid for 3 min, rinsed in 70% ethanol, dehydrated with increasing concentrations of EtOH and xylene. Sections were mounted and imaged with the Olympus Slide View. Picrosirius red staining was quantified using ImageJ.

### 4.5 Mouse Tissue Extraction and Dissociation

Mice were euthanized in a CO_2_ chamber, and cervical dislocation was used as a secondary validation of death, as recommended by the IACUC. For liver extraction, perfusion was performed by injecting 5 mL of DPBS (Corning) into the vena cava with a syringe, and the portal vein was cut to allow fluid to drain. Each extracted liver, kidney, or 2–3 lungs pooled together from mice in the same group was briefly washed in PBS, then transferred into an individual gentleMACS C Tube (Miltenyi Biotec) containing 5 mL of appropriate dissociation buffer and minced with scissors. For dissociation of liver and kidney, the dissociation buffer contained 10 mM HEPES, 137 mM NaCl, 1 mg/mL collagenase I (Sigma‐Aldrich), 1% BSA, and 0.04 mg/mL DNase I. For lungs, a dissociation buffer containing 10 mM HEPES, 137 mM NaCl, 2 mg/mL collagenase I (Sigma‐Aldrich), 1% BSA, and 0.04 mg/mL DNase I was used. The gentleMACS C tubes were then attached to the gentleMACS Octo Dissociator with Heaters (Miltenyi Biotec). For lung, the m_lung_01_01 program was run three times, followed by a 30‐min incubation at 37°C on the gentleMACS Octo Dissociator, and 5 s of the program m_lung_02_01. For the kidney, the m_liver_02_02 program was run twice consecutively and incubated for 30 min at 37°C, during which the m_liver_02_02 program was run twice consecutively halfway through incubation. For liver, the m_liver_01_02 program was run twice consecutively and incubated for 30 min at 37°C, during which the m_liver_01_02 program was run twice consecutively halfway through incubation. Cell suspensions were then passed through a 40 μm cell strainer (Corning) and processed with RBC lysis buffer (Zymo Research). For liver, debris removal solution (Miltenyi Biotec) was used following the cell strainer step and before RBC lysis following the manufacturer's instructions.

### 4.6 Rotarod and Open Field

For rotarod, mice were trained three times 2–3 days before measurement. The following setup was used: ramp, initial speed 5 rpm, final speed 40 rpm, ramp 120 s. During training, mice were returned to the rotarod after falling to remain for the total duration. For the measurement, the time of fall was recorded. Measurements were repeated three times and reported as an average for each mouse. For open field (Noldus EthoVision), mice were acclimated for at least 1 h in the testing room. Mice were placed in the EthoVision field of view and spontaneous activity was recorded for 10 min and analyzed using Noldus Ethovision software.

### 4.7 Flow Cytometry and Fluorescence‐Activated Cell Sorting

Single‐cell suspensions of IMR‐90, MEFs, 3T3, mouse lung, kidney, spleen, or liver were stained with 7‐AAD (1:100 dilution) and anti‐Lamp1 antibody (1:100 dilution, human, clone H4A3, BioLegend; mouse, REA777, Miltenyi Biotec) in FACS buffer (PBS with 2% FBS or PBS with 0.5% BSA and EDTA) for 30 min at 4°C, protected from light. After staining, cells were washed with FACS buffer and then analyzed on MACSQuant Analyzer (Miltenyi Biotec) and/or fluorescence‐activated cell sorting on MACSQuant Tyto Sorter (Miltenyi Biotec). To test LAMP1 binding in physiological conditions, trypsinized and washed cells were incubated at 37°C for 15 min with the required antibodies. For the LAMP2 staining, cells were stained at 1:50 for 30 min on ice using LAMP2 Monoclonal Antibody (H4B4) (Invitrogen).

### 4.8 Immunofluorescence

SEN and NS were washed once in PBS and fixed in 4% PFA for 15 min at room temperature. After washing the PFA with PBS 3 times, 0.2% Triton X‐100 was added for 15 min and then washed with PBS three times. Blocking was done using 5% goat serum overnight at 4°C. Primary antibodies (1:500) were prepared in 5% goat serum with TBST and incubated overnight at 4°C. Secondary antibodies (1:1000) and Hoechst (1:2000) were added for 1 h at room temperature in the dark. After washing with PBS, representative images were taken with an EVOS microscope.

### 4.9 Real‐Time Cytotoxicity Assay (xCELLigence RTCA‐MP)

ADC toxicity to senescent IMR‐90 was measured using the xCELLigence RTCA‐MP platform (Agilent). Cytotoxicity was normalized to negative untreated controls, IgG controls, and positive full‐lysis controls. 50 nM of Anti‐Mouse IgG Fc‐Duocarmycin Antibody with Cleavable Linker (MORADEC, AM‐102‐DD) was added to the wells for 2 h after 1 h of anti‐LAMP1 incubation (26 nM). Impedance measurements to assess cell viability were performed every 15 min for 24–48 h. All experiments were performed in triplicates in at least three independent experiments.

### 4.10 Quantitative RT‐PCR

RNA was extracted from mouse tissue cells with Qiagen RNeasy Plus mini kit following the manufacturer's instructions. cDNA was synthesized with PrimeScript RT reagent Kit (Takara Bio) following the manufacturer's instructions. Real‐time PCR was done using AzuraQuant Green Fast qPCR Mix LoRox (RealTimePrimers) on QuantStudio3 (Applied Biosystems, Thermo Fisher) in 96‐well plates, and gene expression levels were calculated using the 2^−ΔΔCt^ method. Unless otherwise specified, Actb was used as the housekeeping control. Mouse *Hprt* was used as the housekeeping control in the BLM‐treated mice lungs.

PCR primers (primer 1; primer 2):

Lmnb1: CAACTGACCTCATCTGGAAGAAC; TGAAGACTGTGCTTCTCTGAGC.

Glb1: CTTCCCACTGAACACTGAGGC; TTGGCACGAACAAGGTCTTTT.

p16: CCGAACTCTTTCGGTCGTAC; AGTTCGAATCTGCACCGTAGT.

p21: TGTCGCTGTCTTGCACTCTG; GACCAATCTGCGCTTGGAGT.

Tgf‐b: TGATACGCCTGAGTGGCTGTCT; CACAAGAGCAGTGAGCGCTGAA.

Hprt: GCTGACCTGCTGGATTACAT; TTGGGGCTGTACTGCTTAAC.

Gapdh: CTGGAGAAACCTGCCAAGTA; TGTTGCTGTAGCCGTATTCA.

Actb: AAGAGCTATGAGCTGCCTGA; TACGGATGTCAACGTCACAC.

### 4.11 Pathway Analysis and Correlation Analysis

To analyze the proteomics screen of the plasma membrane of SEN we used the online tool Enrichr (https://maayanlab.cloud/Enrichr/enrich). The list of 636 genes was obtained from Marin et al. (2023) and a hard threshold was set for upregulation in at least three out of seven senescence induction models (Marin et al. 2023). Enrichr presented as KEGG 2021 Human, BioPlanet 2019 or GO Cellular Component 2023, as indicated in the figure legends. Web interface tool Correlation AnalyzeR was used to study the correlation between LAMP1 and genes of interest, with a “Basic” corGSEA annotation (https://gccri.bishop‐lab.uthSENa.edu/shiny/correlation‐analyzer/).

### 4.12 Single‐Cell RNA Sequencing Analysis

The scRNA‐Seq. data from the “Human Muscle Ageing Cell Atlas (HLMA)” was analyzed using the online tool (https://db.cngb.org/cdcp/hlma/rnaseq/). CDKN1A‐ and LAMP1‐expressing cells were filtered based on > 2 expression to identify cell clusters expressing p21‐high LAMP1‐high (Lai et al. 2024).

### 4.13 Statistical Methods

Graph Pad Prism 10 was used to perform statistical analysis. Images were processed using ImageJ. Flow cytometry data were analyzed using FlowLogic. Gene Set Enrichment Analysis (GSEA) was performed with Galaxy and RNA‐Seq. data quality control was performed by Azenta. Unless otherwise specified, all data are presented as mean ± SEM. Unless otherwise specified, all experiments are representative of at least three independent experiments.

Comparisons between groups were done using an unpaired *t*‐test or ordinary one‐way ANOVA. Statistical parameters are defined in the figure legends.

## Author Contributions

G.M.‐L. and A. Sharma wrote the manuscript. A.Sharma secured the funding for the project, project administration, and supervision. M.J.R. and A.B., extensively reviewed the manuscript. G.M.‐L., M.Q., and A. Sharma designed the study. G.M.‐L. and M.Q. performed most experiments and analyzed results. A.B., B.D., Y.H., A. Boominathan, and A. Shankar performed experiments and analyzed results. All authors participated in discussion and editing the manuscript. All authors have read and agreed to the published version of the manuscript.

## Conflicts of Interest

A patent application related to the work described in this manuscript has been filed by the SENS Research Foundation (SRF), now operating as the Lifespan Research Institute (LRI), with A.S. listed as an inventor. All authors are or have been employees of SRF or LRI and have received salary support from these organizations. The authors declare no conflicts of interest.

## Supporting information


Appendix S1.

Appendix S2.

Appendix S3.

**Figure S1.** LAMP1 expression increases with age and correlates with senescence‐associated genes in healthy and cancer tissues. (a, b) LAMP1 expression in human muscle cells from young and old donors. Data acquired from Muscle Cell Aging Atlas (Lai et al. 2024). (c) LAMP1 expression correlation with CDKN1A (p21) expression in cancer tissue (Correlation AnalyzeR). (d) LAMP1 expression correlation with the senescence surface biomarker uPAR (PLAUR) in healthy tissue (Correlation AnalyzeR).

**Figure S2.** Etoposide‐induced SEN upregulate LAMP1 on the plasma membrane. (a) Representative image of etoposide‐treated SEN and NS IMR‐90 controls after SA‐β‐Gal colorimetric assay. Scale bar = 150 μm. (b) Percentage of LAMP1^+^ cells in total cell population following treatment with etoposide, *n* = 3 biological replicates, ordinary one‐way ANOVA. (c) MFI of etoposide‐treated IMR‐90 fibroblasts stained with LAMP1, *n* = 3 biological replicates, ordinary one‐way ANOVA; data represented as mean ± SEM. ISO = isotype control. (d) Immunoblot assessment of LAMP1 in different subcellular fractions (cell membrane, cytosolic, and lysosomal fractions) of SEN and NS IMR‐90; *n* = 3 biological replicates. (e) Representative flow cytometry plot of cell‐surface LAMP2 in SEN and NS IMR‐90. Left, NS. Right, SEN. Gray represents unstained cells. Black represents isotype controls. ^ns^ *p* > 0.05; **p ≤ 0.01; ***p ≤ 0.001.

**Figure S3.** Lamp1 expression in mouse SEN and organs. (a) Representative flow cytometry histogram of Lamp1 cell‐surface expression in mouse 3T3 cells. Light blue, NS controls. Light red, SEN. Data is representative of *n* = 2 biological replicates. (b) Lamp1^+^ cells in the spleen of middle‐aged mice (39–63 weeks old). Data is representative of *n* = 2 biological replicates. Light gray, unstained. Black, isotype control. Red, Lamp1 expression. (c) Representative flow cytometry gating strategy used to quantify Lamp1^+^ cells in 16‐week‐old mice. Cells were gated based on size, singlets, and viability, and compared to controls. (d) Lamp1^+^ cells in the liver, lungs, spleen, and kidneys of middle‐aged mice (16‐week‐old). Data is representative of *n* ≥ 2 biological replicates. (e, f) Cell fractions sorted for Lamp1 have an increased Lamp1 cell‐surface expression in (e) lungs and (d) liver. Light red, cells sorted based on Lamp1 expression. Data is representative of *n* = 3 biological replicates. (g) Gene expression of *p16*, *p21*, and *Lmnb1* of Lamp1^−^ and Lamp1^+^ cells. Lamp1‐depleted live single cells were used as controls (blue). Data is representative of *n* ≥ 3 biological replicates, unpaired *t*‐test; data represented as mean ± SEM. *Gapdh* used as housekeeping controls. *p < 0.05; ^Ω^ *p* < 0.08.

**Figure S4.** Validation of fibrosis and senescence induction after bleomycin. (a) Left, gene expression of *p21*. *Hprt* was used as a housekeeping control, data represented as mean ± SEM, unpaired *t*‐test. ^&^ *p* < 0.06. Right, gene expression of *p16*. *Gapdh* was used as a housekeeping control, data represented as mean ± SEM, unpaired *t*‐test. (b) Gating strategy used to isolate Lamp1^+^ cells. (c) Representative flow cytometry histogram of Lamp1 expression in saline‐treated controls and BLM‐treated fibrotic lungs. Cd45^+^, Cd11c^−^, and Cd3^+^ cells were considered T cells.

**Figure S5.** Enrichment of senescence‐associated genes in Lamp1‐enriched lung cells. (a) Heat map of the SenMayo genes in Lamp1‐enriched and Lamp1‐depleted cells from sham and BLM mice. *Z*‐score.

Data S1.

Data S2.

## Data Availability

Supporting source data are available online with this manuscript. Data are also available upon reasonable request. The Bulk RNA‐Sequencing data generated in this manuscript has been deposited under the accession GSE297723.
