# Supplementary figures and images for: Cell‐Surface LAMP1 is a Senescence Marker in Aging and Idiopathic Pulmonary Fibrosis
Source: Aging Cell. 2025 Jun 22;24(9):e70141. doi: 10.1111/acel.70141 (PMC12419843; doi:10.1111/acel.70141)

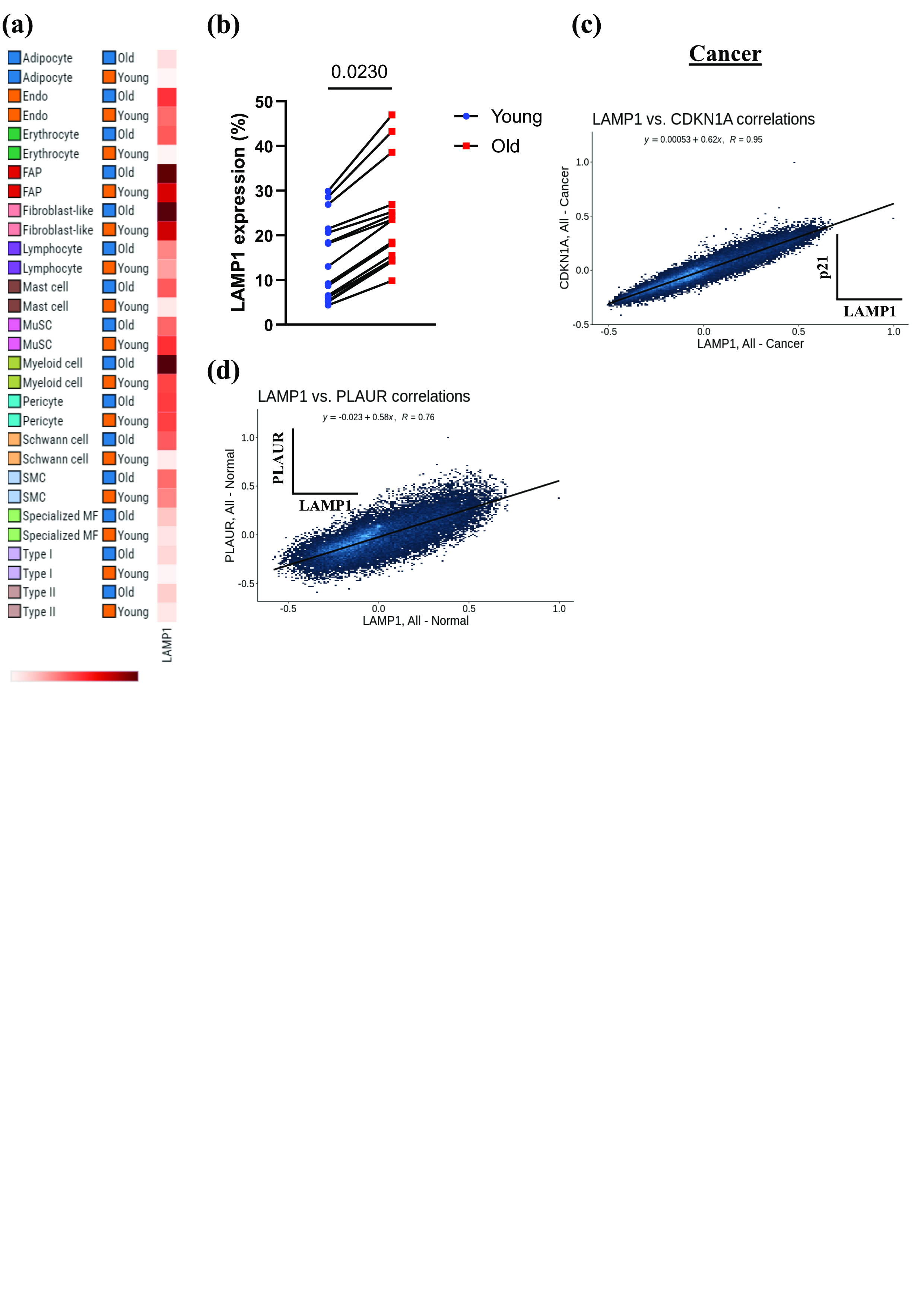

Supplement: Supplementary file 4 — Figure S1. LAMP1 expression increases with age and correlates with senescence‐associated genes in healthy and cancer tissues. (a, b) LAMP1 expression in human muscle cells from young and old donors. Data acquired from Muscle Cell Aging Atlas (Lai et al. 2024). (c) LAMP1 expression correlation with CDKN1A (p21) expression in cancer tissue (Correlation AnalyzeR). (d) LAMP1 expression correlation with the senescence surface biomarker uPAR (PLAUR) in healthy tissue (Correlation AnalyzeR). [file ACEL-24-e70141-s010.tiff]

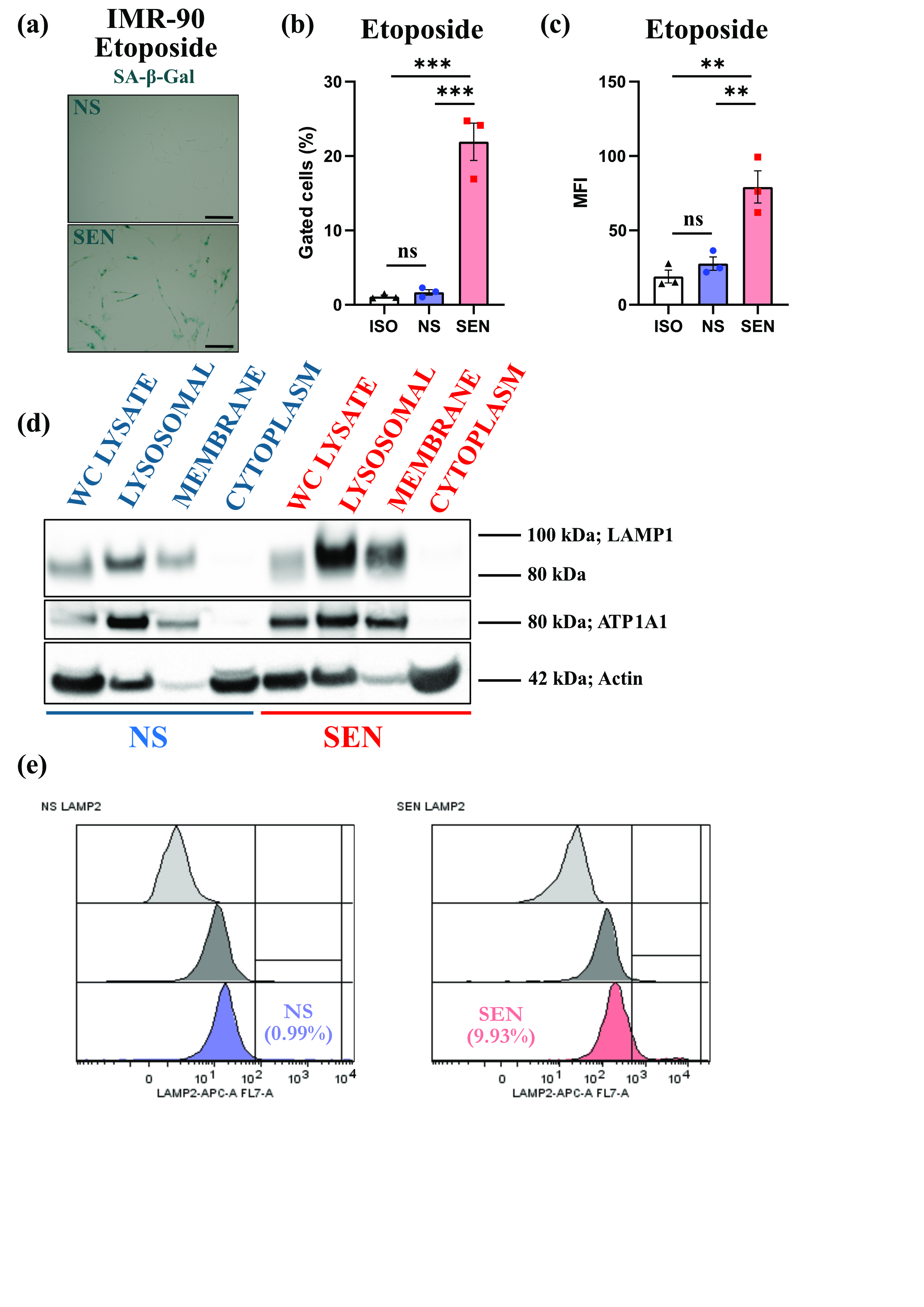

Supplement: Supplementary file 5 — Figure S2. Etoposide‐induced SEN upregulate LAMP1 on the plasma membrane. (a) Representative image of etoposide‐treated SEN and NS IMR‐90 controls after SA‐β‐Gal colorimetric assay. Scale bar = 150 μm. (b) Percentage of LAMP1+ cells in total cell population following treatment with etoposide, n = 3 biological replicates, ordinary one‐way ANOVA. (c) MFI of etoposide‐treated IMR‐90 fibroblasts stained with LAMP1, n = 3 biological replicates, ordinary one‐way ANOVA; data represented as mean ± SEM. ISO = isotype control. (d) Immunoblot assessment of LAMP1 in different subcellular fractions (cell membrane, cytosolic, and lysosomal fractions) of SEN and NS IMR‐90; n = 3 biological replicates. (e) Representative flow cytometry plot of cell‐surface LAMP2 in SEN and NS IMR‐90. Left, NS. Right, SEN. Gray represents unstained cells. Black represents isotype controls. ns p > 0.05; **p ≤ 0.01; ***p ≤ 0.001. [file ACEL-24-e70141-s004.tiff]

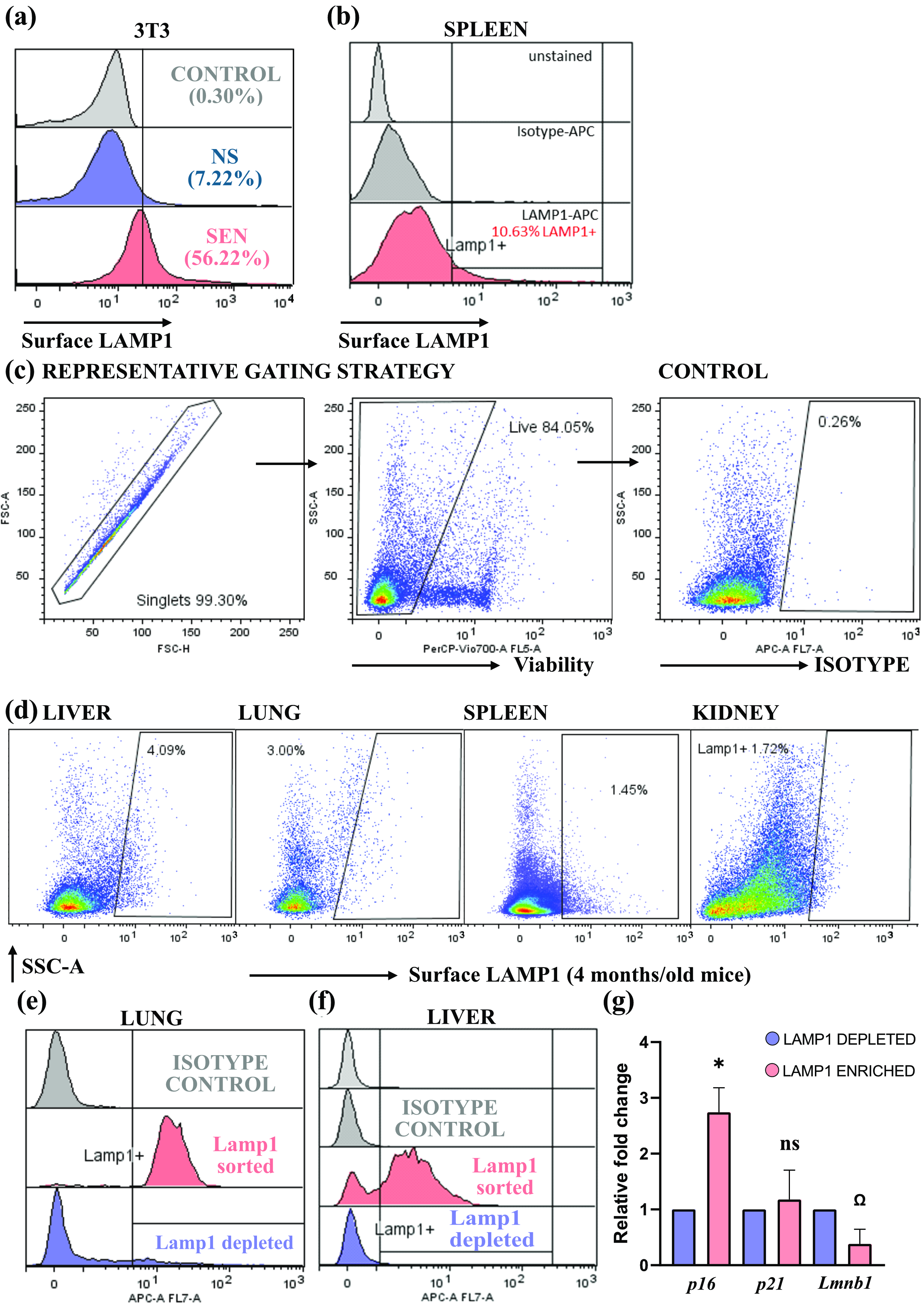

Supplement: Supplementary file 6 — Figure S3. Lamp1 expression in mouse SEN and organs. (a) Representative flow cytometry histogram of Lamp1 cell‐surface expression in mouse 3T3 cells. Light blue, NS controls. Light red, SEN. Data is representative of n = 2 biological replicates. (b) Lamp1+ cells in the spleen of middle‐aged mice (39–63 weeks old). Data is representative of n = 2 biological replicates. Light gray, unstained. Black, isotype control. Red, Lamp1 expression. (c) Representative flow cytometry gating strategy used to quantify Lamp1+ cells in 16‐week‐old mice. Cells were gated based on size, singlets, and viability, and compared to controls. (d) Lamp1+ cells in the liver, lungs, spleen, and kidneys of middle‐aged mice (16‐week‐old). Data is representative of n ≥ 2 biological replicates. (e, f) Cell fractions sorted for Lamp1 have an increased Lamp1 cell‐surface expression in (e) lungs and (d) liver. Light red, cells sorted based on Lamp1 expression. Data is representative of n = 3 biological replicates. (g) Gene expression of p16, p21, and Lmnb1 of Lamp1− and Lamp1+ cells. Lamp1‐depleted live single cells were used as controls (blue). Data is representative of n ≥ 3 biological replicates, unpaired t‐test; data represented as mean ± SEM. Gapdh used as housekeeping controls. *p < 0.05; Ω p < 0.08. [file ACEL-24-e70141-s006.tiff]

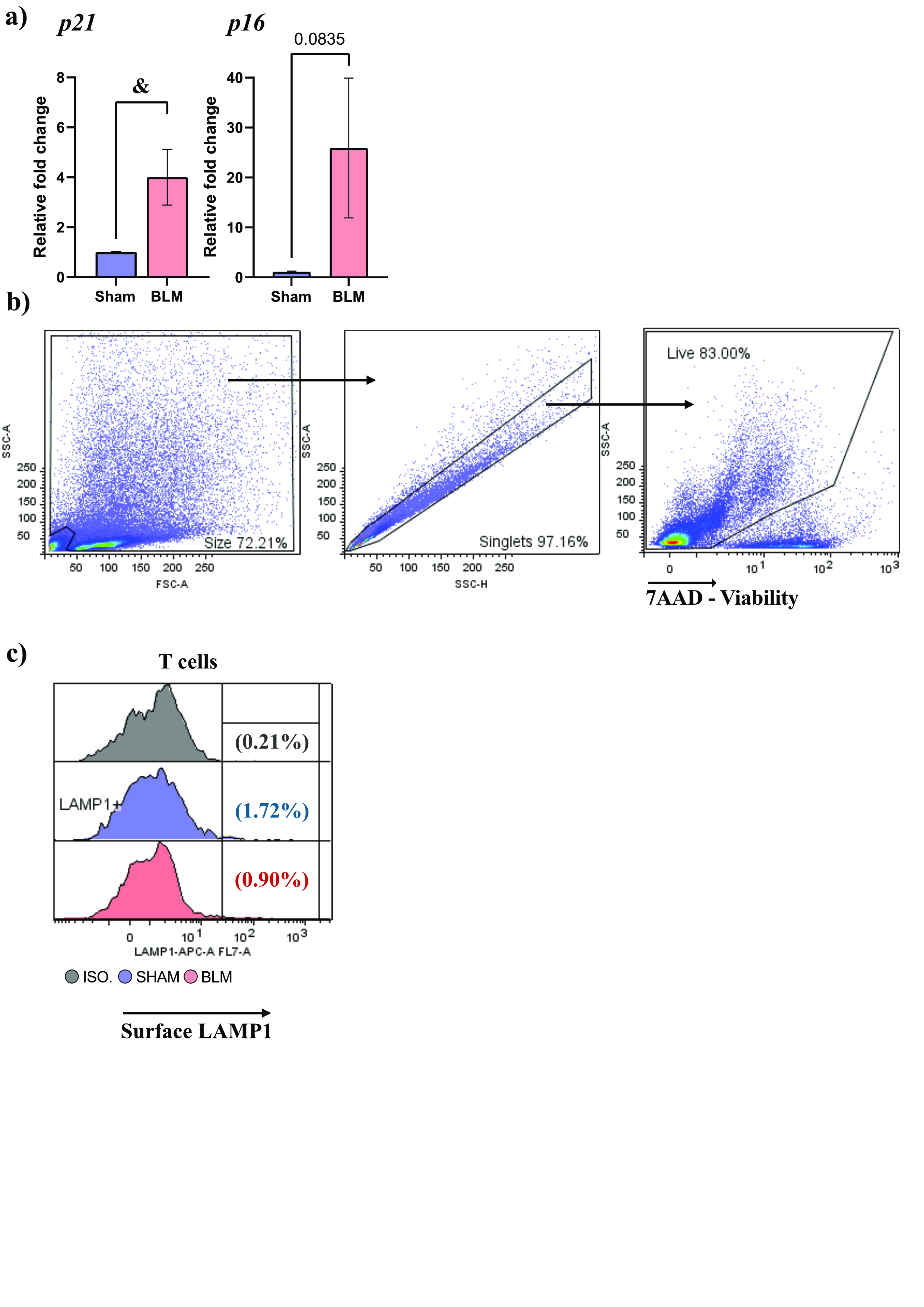

Supplement: Supplementary file 7 — Figure S4. Validation of fibrosis and senescence induction after bleomycin. (a) Left, gene expression of p21. Hprt was used as a housekeeping control, data represented as mean ± SEM, unpaired t‐test. & p < 0.06. Right, gene expression of p16. Gapdh was used as a housekeeping control, data represented as mean ± SEM, unpaired t‐test. (b) Gating strategy used to isolate Lamp1+ cells. (c) Representative flow cytometry histogram of Lamp1 expression in saline‐treated controls and BLM‐treated fibrotic lungs. Cd45+, Cd11c−, and Cd3+ cells were considered T cells. [file ACEL-24-e70141-s003.tiff]

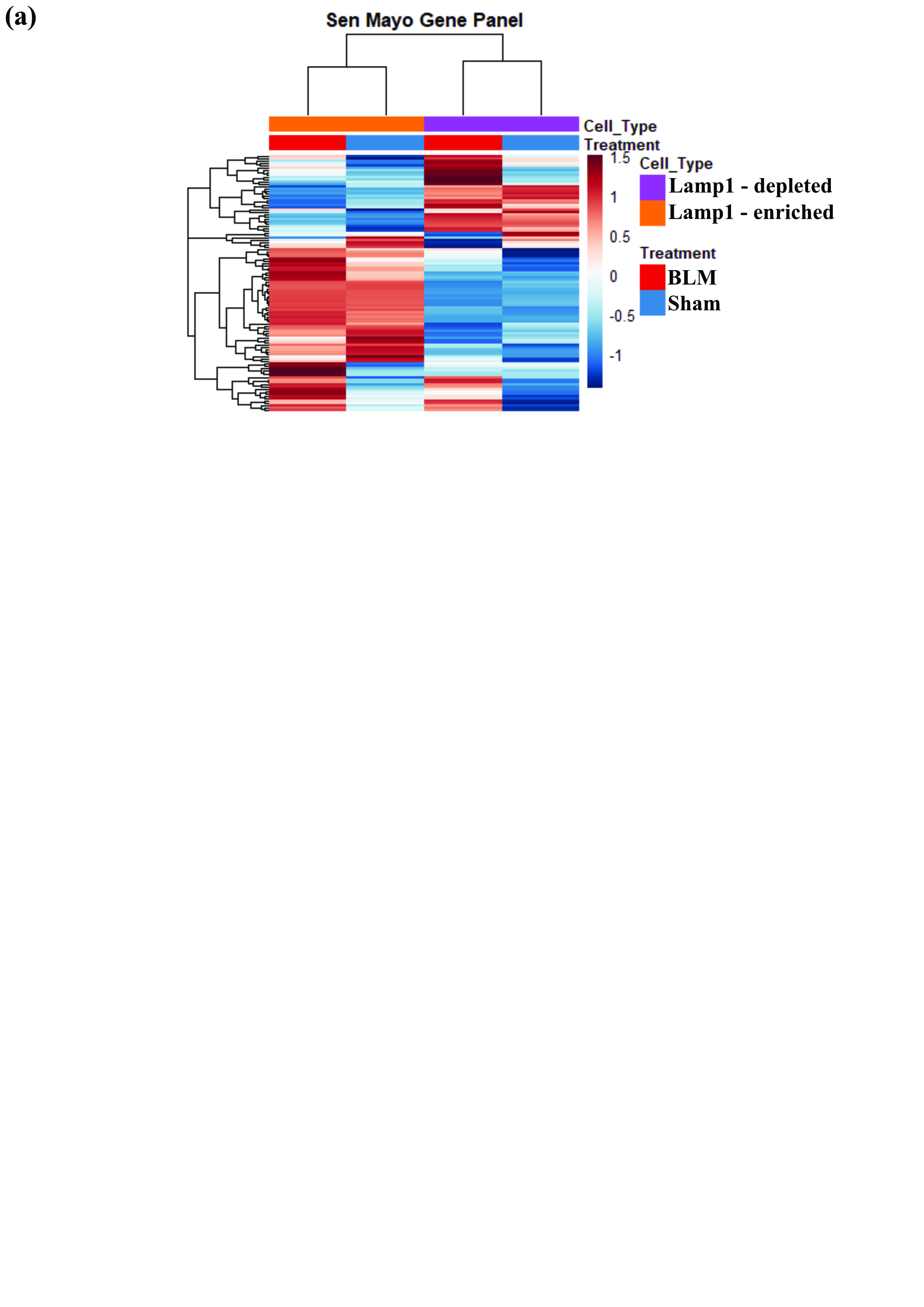

Supplement: Supplementary file 8 — Figure S5. Enrichment of senescence‐associated genes in Lamp1‐enriched lung cells. (a) Heat map of the SenMayo genes in Lamp1‐enriched and Lamp1‐depleted cells from sham and BLM mice. Z‐score. [file ACEL-24-e70141-s005.tiff]
