# Supplementary material for: Cell‐Surface LAMP1 is a Senescence Marker in Aging and Idiopathic Pulmonary Fibrosis
Source: Aging Cell. 2025 Jun 22;24(9):e70141. doi: 10.1111/acel.70141 (PMC12419843; doi:10.1111/acel.70141)
Supplement: Supplementary file 9 — Data S1. [file ACEL-24-e70141-s001.docx]

**Supplementary Methods**

**Subcellular fractionation for LAMP1 protein expression**

Cells were harvested using a cell scraper to preserve membrane protein integrity. For each experiment, approximately 20 million cells were collected and lysed using digitonin (Dixit et al.,2021). A high-speed centrifugation step at 10,000 × g for 10 minutes at 4°C after digitonin lysis yielded a pellet containing mitochondria, lysosomes, and peroxisomes (lysosome-enriched fraction), and a supernatant with cytoplasmic and plasma membrane components. To further isolate the membrane fraction, the supernatant was centrifuged at 50,000 × g for 1 hour at 4°C (Beckman Coulter TLX100), resulting in a pellet corresponding to the membrane fraction and a supernatant containing soluble cytoplasmic proteins. The soluble cytoplasmic proteins were then precipitated with 10% trichloroacetic acid (TCA) containing 1 mM PMSF for 30 minutes on ice, followed by centrifugation at 17,000 × g for 30 minutes at 4°C.

For western blot analysis, the membrane and lysosomal fractions were resuspended in 50 μL of 2× SDS buffer (with 100 mM DTT), while the cytoplasmic pellet was resuspended in 30 μL of 0.5 M Tris-HCl (pH 8.0) to adjust pH and 50 μL of 2× SDS buffer. Equivalent volumes of each fraction (lysosomal, cytosolic and membrane fractions) reflecting equivalent starting cell numbers for senescent and non-senescent samples were loaded on the gel. Whole-cell lysates were prepared separately, normalized for cell numbers and 20–30 μg of protein, as determined by BCA assay, was loaded per lane.

SDS-PAGE was performed on NuPage™ 4–12% Bis-Tris gels using Novex™ Sharp Pre-Stained Protein Standard (Invitrogen, Carlsbad, CA) (Boominathan et al, 2016). Immunoblots were probed with antibodies against LAMP1, ATP1A1, and β-actin (ATP1A1 - Sodium Potassium ATPase Recombinant Rabbit Monoclonal Antibody (Invitrogen; ST0533); Actin - β-Actin Antibody (Cell Signaling; #4967); Exposure times: LAMP1 – 3 seconds; ATP1A1 – 15 seconds; Actin – 30 seconds; primary antibody dilutions: LAMP1 – 1:1000; ATP1A1 – 1:2000; Actin – 1:1000; secondary antibody dilutions: 1:3000). Horseradish peroxidase-conjugated secondary antibodies to mouse (cat. no. G21040), rabbit (cat. no. A16023), and goat (cat. no. G21040) was obtained from Life Technologies (Carlsbad, CA).
